# Zero-Shot Classification of Postoperative Complications From Real-World Discharge Letters According to the Clavien-Dindo System Using Large Language Models in Liver Surgery: Comparative Study

**DOI:** 10.2196/92407

**Published:** 2026-08-21

**Authors:** Sina Warmer, Kamyar Arzideh, Marie Morys, Ahmad Idrissi-Yaghir, Jan Bednarsch, Daniel Heise, Arzu Oezcelik, Marc Reschke, Tom F Ulmer, Ulf P Neumann, Felix Nensa, Katarzyna Borys, René Hosch, Sophia M Schmitz

**Affiliations:** 1 Institute for Artificial Intelligence in Medicine (IKIM) University Hospital Essen Essen Germany; 2 Institute of Diagnostic and Interventional Radiology and Neuroradiology University Hospital Essen Essen Germany; 3 Department of General, Visceral, Vascular and Transplantation Surgery University Hospital Essen Essen Germany; 4 Department of Surgery Maastricht UMC+ Maastricht The Netherlands

**Keywords:** large language models, Clavien-Dindo classification, AI in health care, postoperative complications, natural language processing, health care interoperability

## Abstract

**Background:**

The standardized extraction of postoperative complications from unstructured routine clinical documentation remains a major unresolved challenge in digital surgery and health informatics. Although the Clavien-Dindo classification is the established standard for grading postoperative complications, its application in routine clinical documentation is largely implicit and unstructured, limiting scalable quality assessment in surgical care.

**Objective:**

This study aimed to assess the capability of open-weight and proprietary large language models (LLMs) to classify postoperative complications according to the Clavien-Dindo system using discharge letters, benchmarked against expert annotation.

**Methods:**

We analyzed discharge letters from 650 surgical cases of 649 patients (median 67, IQR 58-73 y; 229/649, 35% female) who underwent hepatobiliary surgery between 2010 and 2024. The cohort included grade I-II complications in 24% (153/650), grade III-IV in 19% (121/650), and grade V (death) in 6% (42/650) of patients. A total of 4 open-weight (Qwen3-235B [Alibaba Cloud], Llama-3.3-70B [Meta AI], GPT-OSS-120B [OpenAI], Ministral-3-8B [Mistral AI]) and 2 proprietary (GPT 5.1 [OpenAI], Gemini 3 Pro [Google]) LLMs were prompted to infer complication grades directly from the discharge letters in a zero-shot setting. Model performance was evaluated against expert assessment using accuracy, *F*_1_-scores, and Cohen κ. To assess interrater reliability and establish a human benchmark, a stratified 10% (n=65) subset was independently annotated by a second clinician, and Cohen κ was calculated between annotators and between each model and the primary expert.

**Results:**

Interrater agreement between the 2 clinical annotators yielded a Cohen κ of 0.75, providing a human benchmark for model performance interpretation. On the full 650-case dataset, open-weight models achieved accuracies ranging from 0.75 to 0.78 for fine-grained prediction, with weighted *F*_1_-scores of 0.76-0.78 and macroaveraged *F*_1_-scores of 0.50-0.63. For binary classification, accuracies ranged from 0.93 to 0.94, with weighted *F*_1_-scores of 0.93-0.95 and Cohen κ of 0.76-0.79, approaching the human interrater benchmark. On a balanced 50-case subset, used as the sole basis for direct cross-model comparison, proprietary models achieved accuracies of 0.78 for fine-grained and 0.94-0.98 for binary classification. An ensemble approach yielded additional gains in classification performance.

**Conclusions:**

LLMs demonstrated promising accuracy in classifying postoperative complications from discharge letters in a zero-shot setting, with performance approaching the upper bound of human interrater agreement. Open-weight models offer a particularly attractive trade-off between accuracy and computational efficiency, while ensemble strategies further enhance robustness. These results support the potential of LLMs to standardize complication assessment at scale and enable data-driven quality monitoring in surgical care.

## Introduction

The automated extraction of clinically meaningful information from routine medical unstructured documentation has become a central ambition of digital medicine and health informatics [1-4]. The standardized classification of postoperative complications represents a particularly relevant and challenging benchmark. Over recent decades, the Clavien‑Dindo classification has emerged as the most widely used system for stratifying surgical complications by severity [5]. Originally proposed by Pierre‑Alain Clavien and Daniel Dindo in 2004 [6], the system grades complications based on their therapeutic consequences from grade I (any deviation from the normal postoperative course without need for pharmacologic, endoscopic, or radiologic intervention) through grade V (death of the patient). Its consistent application is important for surgical quality assurance, benchmarking, and outcome research. Additionally, its usage in randomized controlled surgical trials has strongly increased in the last decades [5]. Yet, in everyday clinical workflows, complications are often recorded implicitly within unstructured text, such as operative notes, discharge letters, or nursing reports. This lack of structured representation hinders large-scale outcome monitoring and perpetuates bias through manual abstraction or selective reporting.

Large language models (LLMs) have recently emerged as powerful tools for reasoning over unstructured clinical narratives, offering the potential to bridge this gap [7-10]. These models demonstrate promising performance in natural language understanding, contextual inference, and classification [11,12], all without the need for manual feature engineering. However, the clinical adoption of LLMs must be considered in the context of data privacy, transparency, reproducibility, and interoperability. The growing ecosystem of open-weight and proprietary language models offers distinct trade-offs across these dimensions. Proprietary frontier models (eg, GPT-5 [OpenAI], Claude [Anthropic], Gemini [Google]) often achieve superior general reasoning but remain opaque and unsuitable for sensitive medical data due to closed architectures and uncertain data provenance [13,14]. In contrast, open-weight models (eg, Llama 3 by Meta AI [15], Mistral by Mistral AI [16], Qwen by Alibaba Cloud [17], GPT-OSS by OpenAI [18]) support local deployment and fine-tuning, enabling compliance with data protection regulations such as GDPR (General Data Protection Regulation) and the broader requirements of the European Union AI Act, while fostering reproducible, auditable research [19-21]. Small language models (SLMs) and domain-specific variants further enable resource-efficient deployment within hospital infrastructures [22].

In this study, we systematically evaluate and compare open-weight and proprietary LLMs for automatic Clavien-Dindo classification using only existing discharge letters from routine clinical documentation. Leveraging real-world surgical data, we examine each model’s ability to infer complication grades from unstructured text and quantify accuracy, consistency, and explainability. Moreover, we introduce a downstream interoperability layer that maps model outputs to Fast Healthcare Interoperability Resources (FHIR) [23]–compatible structured representations, thus bridging the gap between unstructured text interpretation and structured, machine-readable outcome documentation.

## Methods

### Ethical Considerations

This study was approved by the Ethics Committee of the Medical Faculty of the University of Duisburg-Essen (approval 23-11557-BO). Due to the study’s retrospective nature, the requirement of written informed consent was waived by the ethics committee. All data were fully anonymized before being included in the study.

### Dataset Collection

**Figure 1:**
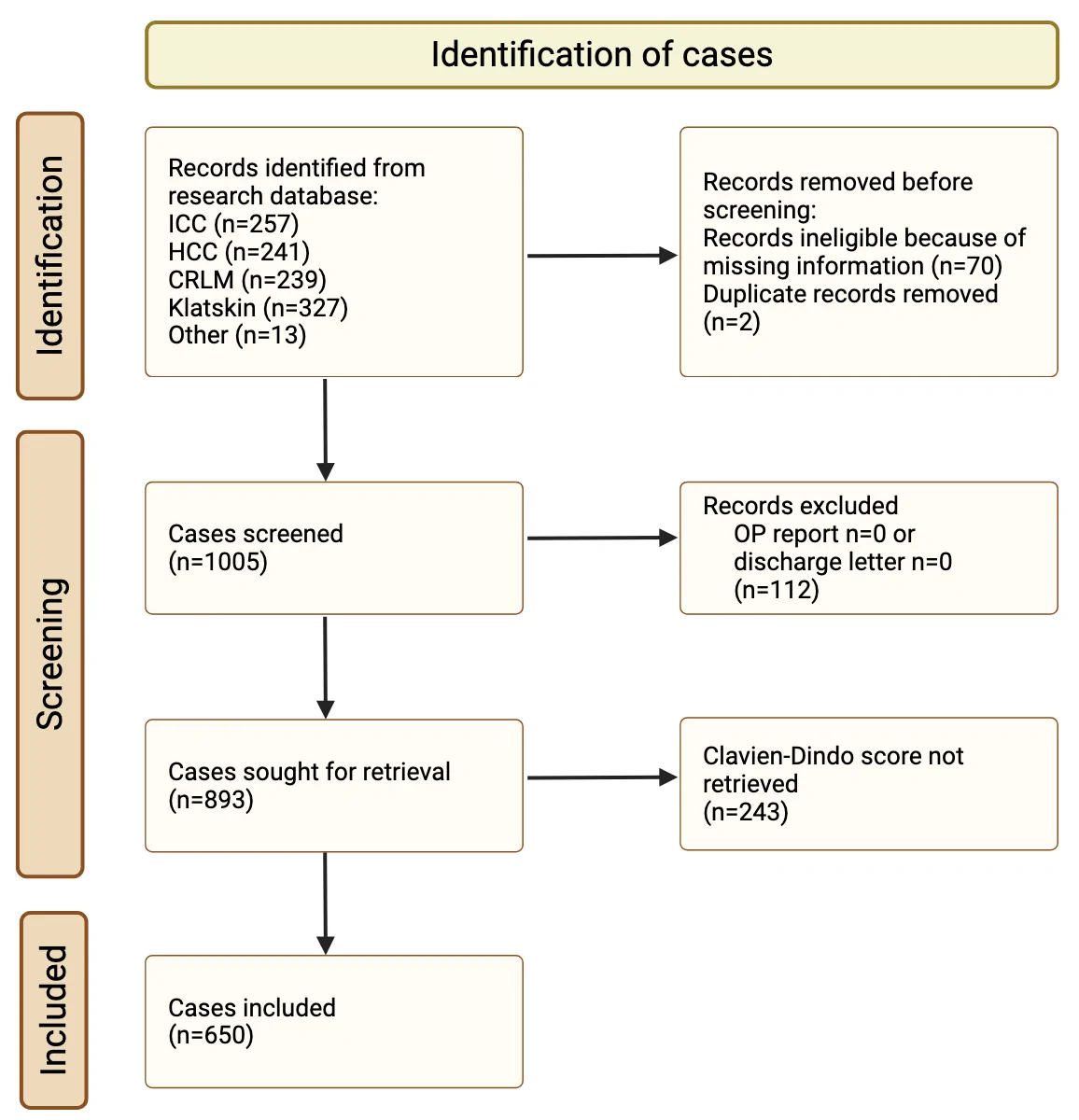
Flow diagram illustrating the stepwise identification of the study cohort. The figure shows successive screening and exclusion steps, resulting in a reduction from 1077 postoperative cases extracted from the hospital information system to 650 eligible cases with expert-assigned Clavien-Dindo grades used for model evaluation (created with BioRender). CRLM: colorectal liver metastases; HCC: hepatocellular carcinoma; Klatskin: perihilar cholangiocarcinoma; ICC: intrahepatic cholangiocarcinoma.

Patients were eligible for inclusion if they had a liver resection between 2010 and 2024, their electronic health record contained at least 1 operative report confirming a hepatic surgical procedure, and at least 1 discharge letter documenting the perioperative course. Across all hepatobiliary service lines—hepatocellular carcinoma (HCC), colorectal liver metastases (CRLM), perihilar cholangiocarcinoma (Klatskin tumors), and intrahepatic cholangiocarcinoma (ICC)—a total of 650 postoperative cases met these criteria and were included after preprocessing (Figure 1).

For each patient, all available discharge and transfer letters were initially retrieved. As part of the expert annotation workflow, the final surgical discharge letter was manually selected for every patient. This expert-selected physician letter was treated as the authoritative postoperative summary and served as the sole input document for model prompting. During data preprocessing, letterhead sections were removed to standardize the narrative input.

### Expert Annotation

**Figure 2:**
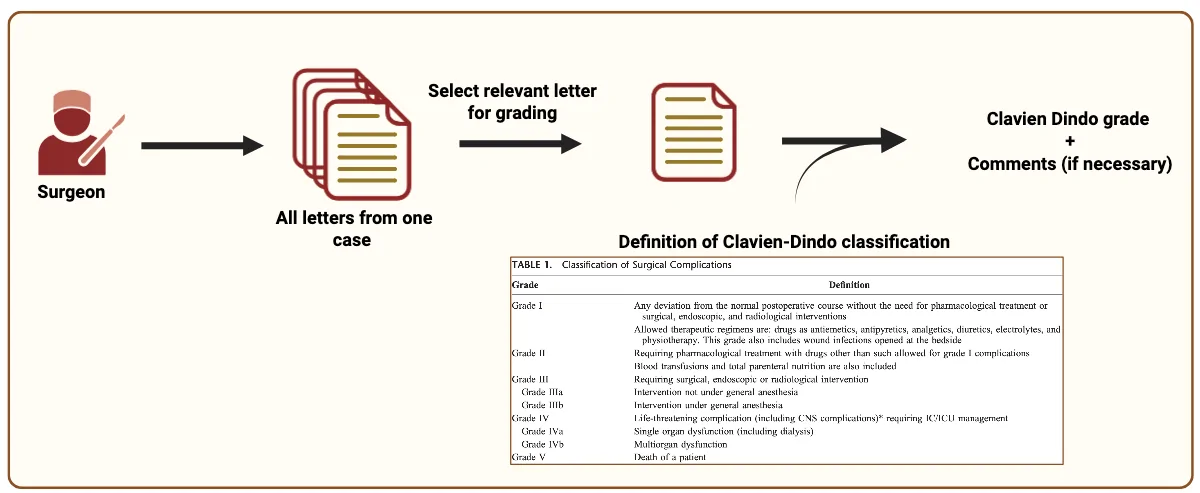
Expert annotation workflow for Clavien-Dindo grading. Schematic overview of the annotation process. For each case, the annotating surgeon reviewed all available discharge letters and selected the clinically most relevant document. Based on this letter and a standardized Clavien-Dindo definition displayed alongside, a complication grade was assigned. Optional free-text comments could be added to document uncertainty or case-specific considerations (created with BioRender).

Expert labeling was performed by a senior attending surgeon with >10 years of clinical experience. For each case, the annotator was presented with all available discharge, transfer, and investigation letters from the relevant encounter, from which the surgical discharge letter was selected. The Clavien-Dindo grade was assigned exclusively based on the content of the selected letter, without access to additional clinical information such as laboratory values, imaging reports, or intraoperative findings, to ensure strict alignment with the information available to the language models (Figure 2).

The manual annotation process was guided by a designated annotation user interface, which presented concise definitions of all Clavien-Dindo grades. Annotators assigned a single, predefined grade for each case using this structured interface, ensuring standardized input and minimizing formatting variability. Free-text comments could be added to flag ambiguous or borderline cases, facilitating subsequent review and, when necessary, refinement of the consensus labels. The resulting expert labels were used as the reference ground truth for all subsequent analyses.

### Interrater Reliability Assessment

To assess the reliability of the expert annotation and to establish a human benchmark for model performance evaluation, a stratified random subset of 65 (10% of the full dataset) cases was selected for independent second annotation. Cases were sampled to approximate a balanced grade distribution across all Clavien-Dindo grades. Grades I-V were each represented by 8 cases. An exception was made for grade IVb, which occurred only 6 times in the full dataset and was therefore included in its entirety. Grade 0 was represented by 11 cases, reflecting its proportionally higher prevalence in the overall cohort.

The second annotation was performed by an independent senior surgeon with 9 years of experience in hepatobiliary surgery and established familiarity with the Clavien-Dindo classification system. Both annotators were blinded to each other’s ratings, and the second annotator had no access to the primary expert annotations during the review process. Interrater agreement was quantified using Cohen κ, which accounts for agreement beyond chance. The resulting κ value serves as a human reference benchmark, allowing model-to-expert agreement, also reported as Cohen κ throughout the evaluation, to be interpreted in the context of clinician-to-clinician variability inherent to Clavien-Dindo grading.

### Model Selection

For this study, a diverse set of LLMs was selected, encompassing both open-weight and proprietary architectures to assess performance across different model classes and deployment constraints (Table 1). Throughout this manuscript, the term “open-weight” refers to models whose trained parameters are publicly accessible and can be deployed locally, but whose training data, code, or full technical specifications may not be fully disclosed, distinguishing them from strictly “open-source” models, which imply complete transparency across all components. For the open-weight category, we selected 4 current LLMs: Qwen3 (Alibaba Cloud), Llama 3.3 (Meta AI), GPT-OSS (OpenAI), Ministral 3 (Mistral AI). These models were chosen for their strong general-purpose reasoning capabilities, broad community support, and the ability to deploy them locally, ensuring full compliance with institutional data protection regulations. Local deployment also enabled deterministic control over inference settings and resource allocation.

**Table 1:** Large language models evaluated in this study. Qwen3, Llama 3.3, GPT-OSS, and Ministral 3 were locally deployed, while GPT-5.1 and Gemini 3 Pro were accessed via commercial APIs.

Model name	Hardware specifications	Open-weight
Qwen/Qwen3-235B-A22B-Instruct-2507-FP8	4x NVIDIA H100 GPU^a^	Yes
meta-llama/Llama-3.3-70B-Instruct	1x NVIDIA A100 GPU	Yes
openai/gpt-oss-120B	1x NVIDIA H100 GPU	Yes
mistralai/Ministral-3-8B-Instruct-2512	1x NVIDIA RTX A6000 GPU	Yes
gpt-5.1-2025-11-13	Commercial deployment	No
gemini-3-pro-preview	Commercial deployment	No

^a^GPU: graphics processing unit.

To contextualize performance, we additionally included the 2 state-of-the-art proprietary models: GPT-5.1 and Gemini 3 Pro. These models were accessed via secure API end points (provider: OpenAI API) and selected for their strong benchmark performance in clinical reasoning and information extraction tasks [24-27].

### Prompting Approach

Model interaction was based on a structured prompting framework, which we refined iteratively in consultation with the surgical expert to optimize clarity, task decomposition, and alignment with the Clavien-Dindo classification criteria. All evaluations were conducted in a zero-shot setting, without task-specific fine-tuning or exposure to labeled examples, to assess the intrinsic out-of-the-box capability of each model to infer postoperative complication severity directly from unstructured clinical documentation. This approach was deliberately chosen to establish a reproducible baseline that reflects the models’’ general reasoning ability rather than optimized task-specific performance. The final prompt consisted of 4 sequential components: a concise task description, a definition of the Clavien-Dindo scale, instructions for determining the correct grade, including a required output schema, and the full physician letter for each case. This structure ensured that models received identical contextual and instructional information. Full prompt templates are provided in Supplement S1-S4 in Multimedia Appendix 1.

**Figure 3:**
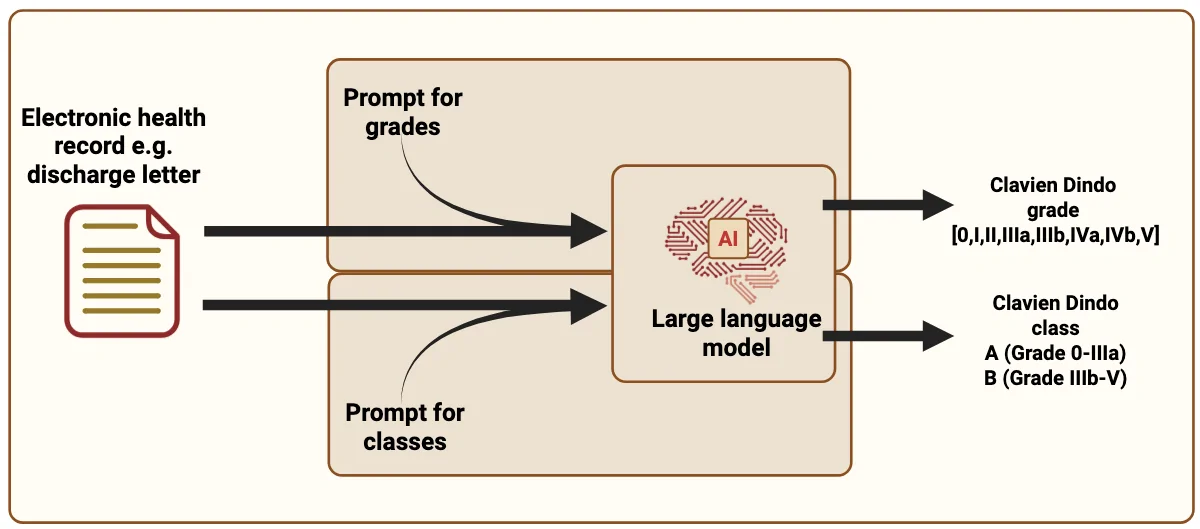
Large language model–based assessment of postoperative complications from electronic health records. Overview of the 2 evaluation scenarios applied to postoperative discharge letters. The upper part of the figure illustrates fine-grained prediction of Clavien-Dindo grades, while the lower part depicts binary classification into minor (≤IIIa) and major (≥IIIb) complication categories based on the same electronic health record inputs (created with BioRender).

Prompting was conducted under 2 distinct scenarios (Figure 3). In the first scenario, models were asked to predict the fine-grained Clavien-Dindo grade (0-V). In the second scenario, we derived a binary classification scheme in which the grades were grouped as *minor complications* (grades 0-IIIa) and *major complications* (grades IIIb-V). For each model output, we extracted 3 predefined fields, including score or category, reason, and citation, to enable structured downstream analysis. The same prompt was applied to all models without model-specific modifications. Additional context length constraints varied across models, and the Llama-3.3-70B was limited to approximately 6000 tokens, requiring truncation of a few excessively long physician letters. In such cases, appendices without additional clinical content were removed after manual review to retain the document’s clinically relevant core. All further hyperparameters are listed in Table S1 in Multimedia Appendix 1.

### Evaluation of Model Performance

**Figure 4:**
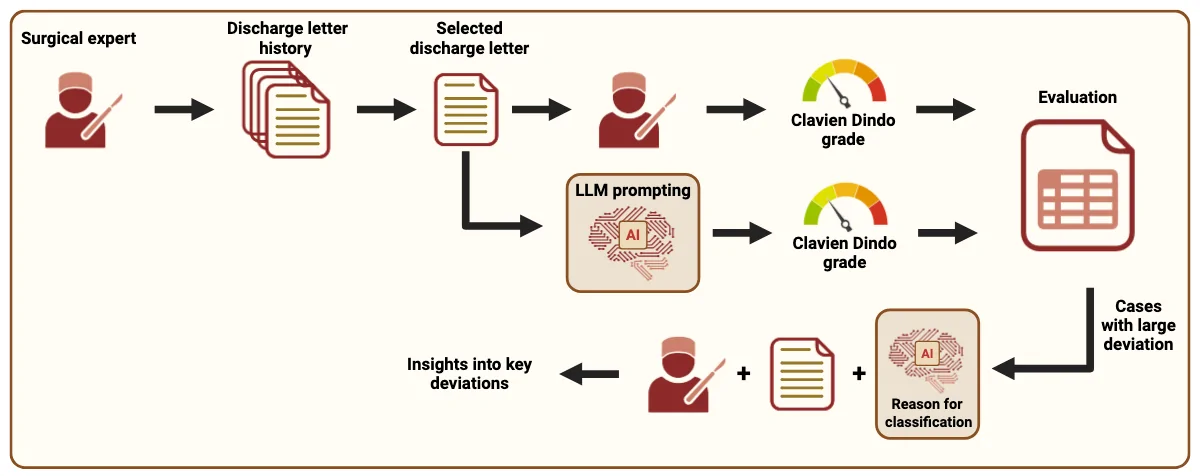
End-to-end evaluation workflow for Clavien-Dindo classification. Schematic overview of the complete study pipeline, including expert annotation of postoperative discharge letters, structured prompting of large language models, quantitative performance evaluation, and manual review of cases with large prediction deviations for error analysis (created with BioRender). LLM: large language model.

Model performance was assessed by comparing the Clavien-Dindo grades predicted by each LLM with the expert-assigned ground truth labels. Evaluation metrics included overall accuracy, grade-specific accuracy, entity-specific accuracy, precision, recall, *F*_1_-scores (per grade, weighted *F*_1_ and macro *F*_1_), and several measures of prediction error relative to expert grading. Accuracy, defined as the model’s agreement with the expert annotation, was calculated both across all cases and stratified by Clavien-Dindo grade, allowing assessment of whether model performance varied with complication severity. To account for potential heterogeneity across clinical indications, accuracy was also computed separately for each disease entity (HCC, CRLM, Klatskin tumors, and ICC). For this simplified task, we calculated overall and entity-specific accuracies, along with their corresponding confusion matrices, using ground-truth categories derived directly from the expert’s assigned Clavien-Dindo grades (Figure 4).

To quantify the magnitude of model-expert-disagreement, we calculated the absolute deviation, defined as the number of grade steps by which a model’s prediction differed from the ground truth. We report the distribution of deviation magnitudes, including the proportion of predictions differing by 1, 2, or more grade levels. These analyses were conducted overall and within each Clavien-Dindo grade, thereby enabling identification of grades that were particularly prone to over- or underestimation. In a final review step, outlier cases were defined as predictions deviating by more than 2 Clavien-Dindo grades from the expert-assigned reference and were manually reviewed. These cases were analyzed qualitatively to identify recurrent sources of disagreement, such as ambiguous documentation, implicit references to complication management, and inconsistent phrasing in physician letters. In addition, a majority voting approach was applied, aggregating predictions across the evaluated models and selecting the final grade based on the most frequent prediction. This ensemble strategy was used to assess whether combining model outputs could improve overall accuracy and reduce extreme disagreement with the expert annotation, under the assumption that individual models exhibit partially complementary error patterns, as suggested by the preceding error and deviation analyses.

### Proprietary Subset Selection

For evaluating proprietary LLMs, a deidentified subset of 50 cases was created. This subset was intentionally balanced across the Clavien-Dindo grades, with 24 cases representing minor (0-IIIa) and 26 cases representing major (IIIb-V) complications. All documents underwent systematic deidentification [28], replacing patient names, dates, institutional references, and location information in alignment with institutional data protection guidelines. This subset was used exclusively for evaluating GPT-5 and Gemini 3 Pro, whereas open-weight models were evaluated on the full dataset of 650 cases.

### Statistical Analysis

Continuous variables were summarized as medians with IQRs. Before correlation analysis, normality of continuous variables was assessed using the Shapiro-Wilk test. As variables did not meet the assumption of normality, continuous clinical variables (eg, length of stay [LOS]) were analyzed descriptively and explored for association with complication severity using nonparametric rank-based correlation (Spearman ρ), given the ordinal structure of the Clavien-Dindo scale. To investigate potential sources of disagreement between model and expert annotation, logistic regression analyses with classification correctness (correct vs incorrect prediction) as the binary outcome variable were conducted. Document length, measured both in characters (per 1000 characters) and words (per 100 words), was included as the independent variable. Separate models were fitted for each evaluated LLM. Regression coefficients were exponentiated to yield odds ratios (ORs) with corresponding 95% CIs, quantifying the change in the odds of correct classification associated with incremental increases in document length. Statistical significance of individual predictors in logistic regression was evaluated using the Wald *z* test, and the significance level was set to *P*<.05 for all analyses. Interrater agreement between the 2 independent clinical annotators, as well as agreement between each model and the primary expert annotation, was quantified using Cohen κ, calculated overall and separately for each Clavien-Dindo grade. κ values were interpreted according to the commonly used benchmarks (<0.20 slight, 0.21-0.40 fair, 0.41-0.60 moderate, 0.61-0.80 substantial, and >0.80 almost perfect agreement). All analyses were conducted in Python 3.12.9 (Python Software Foundation) using the packages *pandas* (2.3.1), *numpy* (2.3.1), and *scipy* (1.16.3), following a prespecified analytical plan to ensure reproducibility.

## Results

### Cohort Characteristics

The final dataset comprised 650 postoperative cases from 649 patients (n=229, 35% female) with a median age of 67 (IQR 58-73) years, treated between 2010 and 2024, and included 819 discharge letters. The cases cover the 4 major hepatobiliary disease entities: HCC (n=156, 24%), CRLM (n=113, 17%), perihilar cholangiocarcinoma (n=211, 33%), ICC (n=162, 25%), and other (n=8, 1%). The resulting expert annotation demonstrated a broad spectrum of postoperative complication severities within the study cohort. Most patients were classified as having no or minor complications, with grade 0 accounting for 334 (51%) cases, followed by grade I (n=90, 14%), grade II (n=63, 10%), and grade IIIa (n=63, 10%). Major complications were less frequent, including grade IIIb (n=41, 6%), grade IVa (n=11, 2%), grade IVb (n=6, 1%), and grade V (n=42, 6%). Overall, minor complications (grades 0-IIIa) comprised 85% (n=550) of the cohort, whereas major complications (grades IIIb-V) accounted for 15% (n=100; Table 2). The cohort characteristics of the 50-case subset are provided in Table S2 in Multimedia Appendix 1.

**Table 2:** Cohort characteristics stratified by Clavien-Dindo grade.

Characteristics		Clavien-Dindo Grade							
		0	I	II	IIIa	IIIb	IVa	IVb	V
Age (y), median (IQR)		66 (56.25-72)	68 (59-72)	71 (63-76)	66 (59-71)	71 (59-75)	65 (58.5-67.5)	72 (67-75.5)	72 (65.25-75.75)
Total cases, n (%)		334 (51)	90 (14)	63 (10)	63 (10)	41 (6)	11 (2)	6 (1)	42 (6)
Total female, n (%)		116 (35)	34 (38)	26 (41)	28 (44)	12 (29)	3 (27)	0 (0)	10 (24)
Total cases per entity, n (%)									
	ICC^a^	90 (27)	30 (33)	11 (17)	16 (25)	7 (17)	3 (27)	1 (17)	4 (9)
	HCC^b^	89 (27)	24 (27)	9 (14)	9 (14)	4 (10)	1 (9)	2 (33)	18 (43)
	Klatskin	78 (23)	25 (28)	30 (48)	31 (49)	20 (49)	6 (55)	1 (17)	20 (48)
	CRLM^c^	71 (21)	11 (12)	12 (19)	6 (10)	10 (24)	1 (9)	2 (33)	0 (0)
	Others	6 (2)	0 (0)	1 (2)	1 (2)	0 (0)	0 (0)	0 (0)	0 (0)
Year span		2010-2024	2010-2024	2010-2024	2011-2024	2012-2024	2013-2024	2017-2023	2011-2024
Text length in characters, median (IQR)		4194 (3567.75-4814.5)	4533.5 (3854-6175.25)	5239 (4243-6305)	5875 (4552.5-6995)	6044 (4438-6971)	7925 (5892-9316.5)	8486 (7162.5-9709)	4805 (4180.75-6934.25)
Text length in words, median (IQR)		506 (419.5-581.75)	553 (459-736.25)	627 (497-776.5)	705 (536.5-865)	733 (504-842)	1007 (696-1103.5)	1050.5 (847-1215)	555 (471-814.5)
LOS^d^ (d), median (IQR)		10 (8-14)	15 (11-21)	14 (11-23)	23 (17-32.5)	22 (19-32)	42 (24-72)	94.5 (42.25-100.25)	11 (7.25-23)

^a^ICC: intrahepatic cholangiocarcinoma.

^b^HCC: hepatocellular carcinoma.

^c^CRLM: colorectal liver metastases.

^d^LOS: length of stay.

The physician discharge letters exhibited high variability in length, with a median of 4558 (IQR 3797-5849.75) characters and 547 (IQR 450-697.75) words. Document length showed a weak-to-moderate positive association with complication severity (Spearman ρ=0.39 for characters and ρ=0.37 for words; both *P*<.001). The median postoperative LOS was 13 (IQR 9-20) days and increased with higher Clavien-Dindo grades, demonstrating a moderate positive correlation with complication severity (Spearman ρ=0.48, *P*<.001). LOS was also moderately correlated with document length (Spearman ρ=0.35 for characters and ρ=0.32 for words; both *P*<.001), indicating that longer, more complex postoperative courses were accompanied by more extensive physician documentation. Given the distinct nature of Clavien-Dindo grade V, additional analyses excluding these cases are provided in Supplement S5 in Multimedia Appendix 1.

### Interrater Reliability

To contextualize model performance relative to human expert variability, interrater agreement between the 2 independent clinician annotations was assessed on the 65-case stratified subset. Overall, Cohen κ indicated substantial agreement between the 2 annotators (κ=0.752) for fine-grading and almost perfect agreement (κ=0.843) for binary grading, providing a human benchmark against which model-to-expert agreement can be interpreted. Grade-level results are presented in Table 3.

**Table 3:** Interrater agreement between 2 independent clinical annotators across Clavien-Dindo grades on a 10% subset (65 cases).

Grade	Cases, n	Interrater agreement Cohen κ
Overall	65	0.752
0	11	1.0
I	8	0.871
II	8	0.681
IIIa	8	0.719
IIIb	8	0.662
IVa	8	0.405
IVb	6	0.476
V	8	0.932

Agreement varied considerably across grades. Perfect agreement was observed for grade 0 (κ=1.000) and almost perfect agreement for grade V (κ=0.932) and grade I (κ=0.871), reflecting the relative clarity of these categories. Substantial agreement was found for grades II, IIIa, and IIIb (κ=0.681-0.719), while grades IVa and IVb showed the lowest interrater concordance (κ=0.405 and κ=0.476, respectively), consistent with the known ambiguity in clinical documentation. These findings are in line with previously reported interrater variability for Clavien-Dindo grading, where comparable levels of agreement for complex complication grades have been observed in urological surgery [29].

### Performance of Open-Weight Models

#### Results for Fine-Grained Prediction

The open-weight models Qwen3-235B, Llama-3.3-70B, GPT-OSS 120B, and Ministral-3-8B were evaluated on the full dataset of 650 cases in a zero-shot setting. Overall accuracy in predicting the fine-grained Clavien-Dindo grade ranged from 0.749 to 0.775, with Qwen3-235B achieving the highest overall accuracy (0.775) among the open-weight models (Table 4). To provide a more comprehensive picture of model performance on the class-imbalanced dataset, macroaveraged and weighted *F*_1_-scores were additionally calculated. Macroaveraged *F*_1_-scores ranged from 0.499 (Ministral-3-8B) to 0.627 (Qwen3-235B), reflecting the greater challenge of correctly classifying underrepresented grades. Weighted *F*_1_-scores, which account for class frequency, ranged from 0.758 to 0.779 and were more closely aligned with overall accuracy. Per-grade precision and recall values are provided in Table S3 in Multimedia Appendix 1.

**Table 4:** Grade-level and overall classification performance for fine-grained Clavien-Dindo grade prediction across 4 open-weight large language models evaluated on the full 650-case dataset in a zero-shot setting. For each Clavien-Dindo grade, the number of cases, accuracy, and F1-score are reported per model. Overall performance is summarized as overall accuracy, macroaveraged F1-score (unweighted mean across all grades, reflecting performance on rare and common grades equally), and weighted averaged F1-score (weighted by class frequency, accounting for the imbalanced grade distribution).

Grade	Cases, n	Qwen3-235B^a^			Llama-3.3-70B^b^			GPT-OSS 120B^c^			Ministral 3-8B^d^	
		Accuracy	*F*_1_-score	Accuracy		*F*_1_-score	Accuracy		*F*_1_-score	Accuracy		*F*_1_-score
0	334	0.904	0.921	0.853		0.896	0.880		0.899	0.961		0.920
I	90	0.5	0.559	0.556		0.553	0.422		0.507	0.322		0.453
II	63	0.667	0.56	0.746		0.531	0.714		0.608	0.629		0.538
IIIa	63	0.571	0.567	0.429		0.551	0.810		0.694	0.587		0.607
IIIb	41	0.780	0.753	0.707		0.707	0.756		0.775	0.659		0.72
IVa	11	0.273	0.222	0.636		0.412	0.182		0.133	0.636		0.341
IVb	6	0.333	0.444	0.167		0.286	0.0		0.0	0.333		0.444
V	42	1.0	0.988	0.976		0.965	0.976		0.976	0.952		0.964

^a^Overall accuracy=0.775, macroaveraged *F*_1_=0.627, and weighted averaged *F*_1_=0.779.

^b^Overall accuracy=0.749, macroaveraged *F*_1_=0.613, and weighted averaged *F*_1_=0.758.

^c^Overall accuracy=0.772, macroaveraged *F*_1_=0.574, and weighted averaged *F*_1_=0.773.

^d^Overall accuracy=0.768, macroaveraged *F*_1_=0.499, and weighted averaged *F*_1_=0.764.

Grade-specific analysis revealed differences across severity levels (Table 4). Performance was highest for grade 0 (accuracy=0.853-0.961; *F*_1_-score=0.896-0.921) and grade V (accuracy=0.952-1.000; *F*_1_-score=0.964-0.988), whereas predictive performance declined for intermediate and less frequent grades, such as grade I (accuracy=0.322-0.556; *F*_1_-score=0.453-0.559), grade IVa (accuracy=0.182-0.636; *F*_1_-score=0.133-0.412), and grade IVb (accuracy=0.000-0.333; *F*_1_-score=0.000-0.444).

Model-specific performance differences were observed across Clavien-Dindo grades. Qwen3-235B demonstrated the most balanced performance, achieving particularly high accuracy for grades 0, IIIb, and V. Llama-3.3-70B performed well for common complications, but showed reduced accuracy for rare, severe events. GPT-OSS-120B achieved high accuracy for grade IIIa (0.810) and demonstrated consistent performance across most grades. By contrast, Ministral-3-8B exhibited greater instability, producing “not classifiable” outputs in 5 cases (omission rate=0.8%), with abstentions distributed across grades 0, II, and IIIa. The impact on overall accuracy was minimal; excluding unclassified cases from the denominator yielded an accuracy of 0.774, while treating them as prediction errors resulted in an adjusted accuracy of 0.768 (Table S4 in Multimedia Appendix 1). Entity-specific accuracy (Table S5 in Multimedia Appendix 1) showed moderate variation across clinical subgroups. Performance was highest in CRLM cases (accuracy range=0.814-0.832), whereas Klatskin cases exhibited the lowest accuracy (0.687-0.768). Accuracy for HCC and ICC cases was comparable across models, with values between 0.735 and 0.827.

To assess potential temporal trends across the 14-year observation period, model performance was additionally stratified by year of discharge letter and is provided in Table S6 in Multimedia Appendix 1. A stratified analysis comparing minor complications (≤grade IIIa) and major complications (≥grade IIIb) demonstrated similar performance across models. The accuracy for minor complications ranged from 0.74 to 0.78, while major complications showed accuracy values between 0.74 and 0.79, indicating that open-weight models maintained relatively stable performance across the complication severity spectrum. Corresponding confusion matrices for the binary grouping (≤IIIa vs ≥IIIb) are provided in Figure S1 in Multimedia Appendix 1.

Cohen κ was calculated between each model’s predictions and the primary expert annotation on the 65-case subset, enabling direct comparison with the previously established human interrater benchmark (κ=0.752). Results are presented in Table 5.

**Table 5:** Model-to-expert agreement quantified as Cohen κ for fine-grained Clavien-Dindo grade prediction across 4 open-weight large language models, evaluated on the stratified 65-case subset. Cohen κ is reported overall and separately for each Clavien-Dindo grade, allowing direct comparison with the human interrater benchmark (κ=0.752) established on the same subset. Negative κ values indicate agreement below chance level. κ values were interpreted as follows: <0.20 slight, 0.21-0.40 fair, 0.41-0.60 moderate, 0.61-0.80 substantial, and >0.80 almost perfect agreement.

Grade	Cases, n	Qwen3-235B	Llama-3.3-70B	GPT-OSS 120B	Ministral 3-8B
Overall	65	0.656	0.588	0.541	0.663
0	11	0.891	0.816	0.781	0.947
I	8	0.925	0.797	0.774	0.745
II	8	0.662	0.502	0.662	0.662
IIIa	8	0.485	0.405	0.693	0.485
IIIb	8	0.743	0.572	0.570	0.572
IVa	8	0.201	0.485	0.021	0.485
IVb	6	0.408	0.266	–0.027	0.486
V	8	0.932	0.857	0.857	0.932

Overall model-to-expert agreement was moderate to substantial for all evaluated open-weight models, with κ values ranging from 0.541 (GPT-OSS) to 0.663 (Ministral 3-8B). All models fell below the overall human interrater benchmark.

Grade-level analysis revealed consistent patterns across models. Agreement was highest for grade 0 (κ=0.781-0.947), grade I (κ=0.745-0.925), and grade V (κ=0.857-0.932), mirroring the pattern observed in human interrater agreement, where these grades were also most consistently classified. Grades II, IIIa, and IIIb showed moderate to substantial agreement across all models (κ=0.405-0.743), comparable with the human interrater agreement for these grades (κ=0.681, κ=0.719, and κ=0.662, respectively). The most challenging grades were IVa and IVb, where agreement was low across all models (κ=0.021-0.485 and κ=−0.027 to 0.486, respectively), with GPT-OSS 120B showing near-zero agreement, indicating performance at chance level for this category, consistent with the relatively low human interrater agreement for these grades as well (κ=0.405 and κ=0.476). Notably, several models approached or exceeded the human interrater benchmark.

#### Results for Binary Grading

In the binary grading task, all open-weight models achieved higher accuracy than in fine-grade prediction. Overall model accuracy ranged from 0.928 (Ministral-3-8B) to 0.94 (Llama-3.3-70B). Although accuracy metrics were closely clustered across models, the *F*_1_-scores provided additional insight into classification robustness. All models achieved *F*_1_-scores between 0.96 and 0.97, indicating a very strong balance between precision and recall in the binary setting. Confusion matrices for the binary classification task demonstrated that disagreement with expert annotation occurred predominantly as category A predicted instead of B, rather than the reverse, reflecting conservative tendencies in model outputs (Figure S2 in Multimedia Appendix 1).

Omission rates varied substantially across models in the binary classification task (Table S7 in Multimedia Appendix 1). While Qwen3-235B, Llama-3.3-70B, and GPT-OSS-120B produced very few or no unclassified outputs (omission rates=0.5%, 0%, and 0.3%, respectively), Ministral-3-8B declined to classify 36 cases (omission rate=5.5%), all falling into category A. When unclassified cases were treated as prediction errors, Ministral-3-8B’s adjusted accuracy dropped from 0.928 to 0.877, representing the largest impact of omission rate on overall performance across all models.

To evaluate whether model performance varied across clinical indications, accuracy was calculated separately for each hepatobiliary disease entity (Table S8 in Multimedia Appendix 1). The observed performance was highest in CRLM cases, with accuracies ranging from 0.965 to 0.991. Similarly, strong performance was observed for HCC (0.949-0.962) and ICC (0.928-0.951) across all models. The “Other” category showed perfect accuracy in 3 of 4 models, though its small sample size limits generalizability. Performance in Klatskin tumors was comparatively lower, with accuracies between 0.883 and 0.905. In addition to quantitative accuracy metrics, we examined how often models were unable to assign any Clavien-Dindo grade. Such “not classifiable” outputs were rare in the larger open-weight models but were markedly more frequent in the smallest model, Ministral-3-8B, which produced 36 unclassified cases. Because these outputs do not correspond to valid Clavien-Dindo categories, they were excluded from grade-level accuracy calculations but included in qualitative error assessment. To assess the potential influence of temporal trends on model performance, accuracy and weighted *F*_1_-scores were additionally calculated per year across the full 2010-2024 observation period (Table S9 in Multimedia Appendix 1).

Extended evaluation metrics for the binary grading task are provided in Table S10 in Multimedia Appendix 1. Per-class analysis revealed a consistent asymmetry across all models: category A (no or minor complication) was classified with high precision (0.979-0.985) but comparatively lower recall, while category B (major complication) showed the inverse pattern, with higher recall (0.889-0.920) but substantially lower precision (0.719-0.772). This reflects a tendency of all models to over-predict grade B, resulting in fewer missed major complications at the cost of more false positives. Macroaveraged *F*_1_-scores ranged from 0.882 (Ministral 3-8B) to 0.897 (Qwen3-235B), and weighted *F*_1_-scores from 0.932 to 0.945, confirming robust overall performance while accounting for class imbalance. Model-to-expert agreement, quantified as Cohen κ, ranged from κ=0.815 (Ministral 3-8B) to κ=0.877 (Qwen3-235B), indicating almost perfect agreement and exceeding the human interrater benchmark of κ=0.843 established for binary annotation.

### Performance of Proprietary Models

To evaluate the performance of proprietary LLMs under similar conditions, GPT-5.1 and Gemini 3 Pro Preview were prompted on a balanced, deidentified subset of 50 cases selected from the full dataset, ensuring uniform representation across all complication severities. While the 4 open-weight models were primarily evaluated on the full 650-case dataset as reported above, their performance was additionally assessed on the same 50-case subset to provide a methodologically equivalent basis for cross-model comparison. The full-dataset results for open-weight models and subset results for proprietary and open-weight models are not directly comparable. The subset-based results for both proprietary and open-weight models are presented side by side in Tables S11-S15 in Multimedia Appendix 1, including grade-level accuracy, *F*_1_-scores, entity-specific accuracy, deviation distributions, and confusion matrices.

GPT-5.1 and Gemini 3 Pro each achieved an overall accuracy of 0.78, with macroaveraged *F*_1_-scores of 0.766 and 0.751, and weighted *F*_1_-scores of 0.774 and 0.761, respectively (Table 6). By comparison, open-weight models achieved overall accuracies of 0.60-0.70 on the same subset, with macroaveraged *F*_1_-scores ranging from 0.557 to 0.673 and weighted *F*_1_-scores from 0.570 to 0.688 (Table S11 in Multimedia Appendix 1).

**Table 6:** Grade-level and overall classification accuracy and F1-scores for fine-grained Clavien-Dindo grade prediction by 2 proprietary large language models (GPT-5.1 and Gemini 3 Pro Preview), evaluated on a balanced, deidentified 50-case subset in a zero-shot setting. Direct comparison with open-weight model performance on the same subset is provided in Table S11 in Multimedia Appendix 1.

Grade	Cases, n	GPT 5.1^a^			Gemini 3 Pro preview^b^	
		Accuracy	*F*_1_-score	Accuracy		*F*_1_-score
0	6	0.833	0.833	1.0		0.923
I	7	1.0	0.933	1.0		0.875
II	5	0.6	0.667	0.6		0.6
IIIa	6	0.833	0.714	0.833		0.833
IIIb	7	0.714	0.714	0.857		0.800
IVa	6	0.667	0.667	0.333		0.444
IVb	6	0.5	0.667	0.5		0.6
V	7	1.0	0.933	1.0		0.933

^a^Overall accuracy=0.78, macroaveraged *F*_1_=0.766, and weighted averaged *F*_1_=0.774.

^b^Overall accuracy=0.78, macroaveraged *F*_1_=0.751, and weighted averaged *F*_1_=0.761.

Moreover, both proprietary models achieved perfect accuracy of 1.0 for grades I and V. The performance for grades IIIa and IIIb was also strong, with values ranging between 0.714 and 0.857 and *F*_1_-scores from 0.714 to 0.833. In contrast, grade II showed mixed performance, with an accuracy of 0.6 and *F*_1_-scores of 0.600-0.667, similar to the results of the open-weight models. For the rarer grades IVa and IVb, greater variability was observed, with accuracies ranging between 0.333 and 0.667 and *F*_1_-scores between 0.444 and 0.667.

Overall, GPT-5.1 and Gemini 3 Pro demonstrated performance that was partly superior, partly comparable, and in some cases lower than the performance of the strongest open-weight models, depending on the complication grade and clinical entity. Both proprietary models showed clear strengths in several grades, including robust performance in grades I, IIIa, IIIb, and V, while exhibiting similar or slightly reduced performance in other grades, particularly some intermediate or low-frequency grades. When complications were grouped into minor (≤IIIa) and major (≥IIIb) categories, both proprietary models outperformed all open-weight models, achieving higher accuracy in distinguishing clinically relevant severity classes (Table S12 in Multimedia Appendix 1). Entity-level performance was consistently high for both proprietary models. Compared with open-weight baselines, proprietary models performed better or at least as well across all entities; particularly strong gains were observed in Klatskin cases, which involve complex postoperative trajectories and narrative heterogeneity (Table S13 in Multimedia Appendix 1). These results indicate that proprietary models generalize more robustly across diverse disease contexts.

In the binary classification task, proprietary models again achieved the highest performance. On the 50-case subset, GPT-5.1 reached an accuracy of 0.98 and a macroaveraged *F*_1_-score of 0.980, with perfect recall for category A (1.00) and perfect precision for category B (1.00). Gemini 3 Pro Preview achieved an accuracy of 0.94 and a macroaveraged *F*_1_-score of 0.940, with a recall of 0.958 for category A and 0.923 for category B. By comparison, open-weight models achieved accuracies of 0.90-0.94 on the same subset, with macroaveraged *F*_1_-scores of 0.900-0.940 (Table S14 in Multimedia Appendix 1). Entity-level binary classification accuracy for GPT-5.1 and Gemini was uniformly high, ranging from 0.8 to 1.0 across all clinical subgroups, confirming that distinguishing minor from major complications is a highly tractable task for state-of-the-art LLMs (Table S15 in Multimedia Appendix 1).

### Analysis of Classification Errors

#### Predictors of Classification Accuracy

To explore potential sources of disagreement with expert annotation, we examined whether characteristics of the input text were associated with prediction errors. Specifically, we fitted a logistic regression model with classification correctness (correct vs incorrect) as the dependent variable and document length measured in both characters and words as independent variables. A detailed summary of the regression coefficients is provided in Table 7.

**Table 7:** Association between discharge letter length and classification accuracy across all evaluated large language models, reported separately for open-weight models (n=650; Ministral-3-8B n=645 due to unclassified outputs) and proprietary models (n=50, balanced subset). Logistic regression results are presented as odds ratios with 95% CIs and *P* values, calculated per 1000 characters and per 100 words.

Model	Cases, n	OR^a^ (95% CI) per 1000 characters	*P* value	OR (95% CI) per 100 words	*P* value
Qwen3	650	0.83 (0.75-0.91)	<.001	0.86 (0.79-0.93)	<.001
Llama 3.3	650	0.90 (0.82-0.99)	.02	0.91 (0.85-0.98)	.02
GPT-OSS	650	0.87 (0.79-0.96)	.005	0.90 (0.83-0.97)	.008
Ministral 3	645	0.86 (0.78-0.95)	.002	0.89 (0.82-0.96)	.003
GPT 5.1	50	0.93 (0.71-1.2)	.57	0.96 (0.78-1.17)	.68
Gemini 3	50	0.73 (0.54-0.99)	.04	0.80 (0.63-1.00)	.049

^a^OR: odds ratio.

The OR quantifies the change in odds of correct classification per unit increase in the predictor variable (1000 characters or 100 words). For the Qwen3 model, an OR of 0.83 indicates that for each additional 1000 characters, the odds of a correct classification decrease by 17%.

Across most evaluated models, increasing document length was associated with lower odds of correct classification. OR below 1.0 indicates that, per incremental increase in text length, the likelihood of a correct Clavien-Dindo assignment decreased. This effect was statistically significant for all open-weight models and for Gemini 3 Pro, whereas no significant association was observed for GPT-5.1, likely due to the limited number of samples in the proprietary-model subset. These findings suggest that longer discharge letters may introduce additional narrative complexity or ambiguity that complicates model interpretation, rather than uniformly providing more discriminative information.

#### Deviation Analysis

To quantify the magnitude of disagreement between model predictions and expert annotations, we computed the absolute deviation defined as the number of grade levels separating the predicted grade from the reference label (Table 8).

**Table 8:** Distribution of absolute grade deviations between model predictions and primary expert annotation for 4 open-weight large language models, evaluated on the full dataset. Absolute deviation is defined as the number of Clavien-Dindo grade levels separating the predicted grade from the reference label, regardless of direction. Only cases with incorrect predictions are included. Values are reported as absolute case counts (n) and relative proportions (%) of all incorrectly classified cases per model.

Absolute deviation	Qwen3-235B, n (%)	Llama-3.3-70B, n (%)	GPT-OSS 120B, n (%)	Ministral-3-8B, n (%)
1	96 (66.21)	117 (71.78)	83 (56.08)	96 (65.75)
2	37 (25.52)	33 (20.25)	42 (28.38)	27 (18.49)
3	7 (4.83)	4 (2.45)	15 (10.14)	12 (8.22)
4	5 (3.45)	8 (4.91)	5 (3.38)	8 (5.48)
5	—^a^	1 (0.61)	3 (2.03)	2 (1.37)
8	—	—	—	1 (0.68)

^a^Not available.

Across open-weight models, 1-step deviations accounted for 55.29% to 71.17%, representing the majority of disagreement with expert annotation. Deviations with 3 or more steps were comparatively rare, occurring in 7.97%-15,75% of prediction errors across models. Grade-specific deviation distributions indicated that grade 0 predictions exhibited the fewest errors, whereas grades II-IVb demonstrated higher variability in error magnitude. Detailed deviation profiles per open-weight model and per grade are shown in Table S16 in Multimedia Appendix 1.

Additionally, the deviation analysis was conducted on the subset with open-weight and proprietary models, revealing systematic differences. While GPT-5.1 and Gemini 3 Pro produced fewer 1-step deviations than most open-weight systems, they showed a higher proportion of 3-step deviations, indicating that although their predictions were often correct, their errors occasionally involved larger jumps in severity. For 2-step deviations, proprietary and open-weight models were broadly similar. However, GPT-OSS 120B represented an exception to this pattern; its deviation distribution was equal to or worse than that of the proprietary models across several grades, with relatively few 1-step deviations and a comparatively high proportion of larger errors. This trend was further confirmed by the per-grade deviation analysis, where GPT-OSS 120B displayed a deviation distribution more similar to that of the proprietary models than to the stronger open-weight systems. This pattern reflects a more polarized deviation distribution in the proprietary models; they were either correct or close in many cases, but when incorrect, they were more likely than open-weight models to misclassify by a larger margin. Full deviation distributions are shown in Tables S17 and S18 in Multimedia Appendix 1.

#### Analysis of Reasons for High Deviation

Cases with high deviation rates (>2 categories) underwent separate manual analysis. Among these, in 10 cases, prolonged intensive care unit (ICU) monitoring without any further therapy was detected as a life-threatening complication (Clavien-Dindo grade≥IVa), and in 7 cases, postoperative intervention was classified as part of the regular oncologic therapy and not as a surgical complication. Renal failure and liver failure without any specific therapy were also among the reasons for high deviation grades. For further deviation causes, refer to Table 9.

**Table 9:** Reasons for large classification errors (≥3 grade deviation) across models. Cases exhibiting high deviations in at least 1 model are included; some cases showed deviations in multiple models.

Reason	Cases, n	Qwen3-235B, n	Llama-3.3-70B, n	GPT-OSS120B, n	Ministral-3-8B, n
Failure to rescue	4	0	4	0	0
Postoperative ERCP^a^	7	5	2	6	5
Liver failure	5	4	3	2	4
Renal failure	4	0	1	1	3
Prolonged ICU^b^ monitoring	10	0	0	6	6
Documentation error	2	2	2	1	2
Pneumonia with mechanical ventilation	2	0	1	1	1
Other	7	1	0	6	2
Total	41	12	13	23	23

^a^ERCP: endoscopic retrograde cholangiopancreatography.

^b^ICU: intensive care unit.

### Majority Vote

To assess whether aggregating predictions across models could improve classification reliability, a simple majority-vote ensemble was constructed. For each case, the most frequently predicted Clavien-Dindo grade among all open-weight models was selected; ties were resolved by assigning the first occurring grade. Only valid numerical predictions were included in the voting process.

The majority-vote ensemble exceeds the performance of all individual open-weight and proprietary models in the fine-grained setting (Table 10). In the binary grading task, the ensemble reached an accuracy of 0.948, outperforming all individual open-weight models and matching the upper range of the proprietary systems. When expanded to include GPT-5.1 and Gemini 3 Pro Preview, the ensemble across all models achieved 0.794, the highest grade-prediction accuracy observed in the study for fine-grained grading, and maintained a high accuracy for binary grading.

**Table 10:** Accuracy of ensemble approaches for fine-grained Clavien-Dindo grade prediction and binary classification. Results shown for open-weight models only and for all models combined.

	Accuracy for fine-grained grading	Accuracy for binary grading
Ensemble open-weights	0.786	0.948
Ensemble open-weights + proprietary models	0.794	0.948

Additionally, ensemble aggregation reduced large disagreement with expert annotation. Whereas 41 cases exhibited deviations of 3 or more Clavien-Dindo grades in individual model predictions, this number decreased to 14 cases when applying majority voting. This reduction indicates that ensemble-based decision-making effectively mitigates extreme errors by leveraging complementary strengths and partially offsetting individual model biases.

## Discussion

### Principal Findings

In this study, contemporary LLMs demonstrated promising accuracy in classifying postoperative complication severity according to the Clavien-Dindo system using routine discharge letters. Performance was consistently strong across open-weight and proprietary models, with substantial improvement for binary classification of minor versus major complications. No single model uniformly outperformed across all dimensions, highlighting the heterogeneous strengths of different architectures and the value of multimodel strategies. While proprietary models achieved the highest overall accuracy on the balanced 50-case subset, open-weight models demonstrated competitive performance with comparatively lower computational requirements, greater deployment flexibility, and enhanced data privacy compliance, which represent critical advantages for clinical implementation. Given the different dataset sizes used for model evaluation, cross-model comparisons are confined to the balanced 50-case subset. Notably, smaller models such as Ministral-3-8B performed comparably to much larger architectures, suggesting that resource-efficient alternatives may suffice for this task. Ministral-3-8B also exhibited a more conservative response strategy, producing the highest number of “not classifiable” outputs when encountering ambiguous information. While the impact on fine-grained classification was negligible, the effect was more pronounced in binary classification, where the accuracy was reduced by 5% when unclassified cases were treated as prediction errors. To contextualize model performance, interrater reliability between 2 independent clinical annotators yielded a Cohen κ=0.752. Model-to-expert agreement ranged from κ=0.541 to κ=0.663 across open-weight models, falling below but approaching this human benchmark, suggesting that LLM-based classification is operating close to the level of inherent clinician-to-clinician variability in Clavien-Dindo grading. Ensemble aggregation improved performance by leveraging complementary error profiles without requiring additional training or fine-tuning.

### Comparison With Previous Work

The observed association between Clavien-Dindo grades and length of hospital stay replicates the gradient originally reported by Dindo et al [6]. This concordance provides evidence for construct validity of the LLM-based scoring system in the present cohort and indicates that automated classification aligns with established clinical patterns. These findings extend previous work on automated complication grading. Staubli et al [30] reported high accuracy (30/31, 96%) using LLMs for Clavien-Dindo classification, but their study relied on clinical scenarios derived from literature rather than original patient documentation. Can et al [31] demonstrated that LLMs can extract structured variables from routine interventional oncology reports in hepatobiliary patients, with proprietary models outperforming open-weight alternatives in accuracy and longitudinal consistency. Our study confirms these patterns using real-world discharge letters, which contain unstructured narratives, variable documentation styles, and clinically relevant ambiguities absent from standardized vignettes. Importantly, when evaluated on the same 50-case subset, our results additionally demonstrate that open-weight models can reach competitive performance for specific tasks such as binary complication classification, suggesting that the performance gap between model classes may be task-dependent.

The Clavien-Dindo classification has become the most widely adopted framework for reporting surgical complications over the past 2 decades. Following amendments in 2009 [32], the authors reported 90% interrater agreement. However, empirical research on interrater agreement has remained limited, particularly regarding the effects of clinical experience, time constraints, or documentation quality. Studies in urological and head-and-neck surgery have reported only moderate inter-observer agreement for Clavien-Dindo scoring [29,33,34]. Dindo et al [6] further demonstrated significant differences in perceived complication severity between patients, nurses, and physicians. The LLMs evaluated in this study achieved higher consistency than reported human interrater reliability in subspecialty settings, suggesting that automated approaches may offer a more reproducible alternative for complication assessment in complex surgical cohorts.

Logistic regression analysis revealed that increasing document length was associated with lower classification accuracy, suggesting that longer discharge letters introduce narrative complexity rather than discriminative clarity. This underscores the value of concise, structured complication documentation for both automated classification and clinical information transfer. Similarly, determining whether postoperative interventions represent surgical complications or planned disease management proved challenging. Updated consensus guidelines now recommend classifying all postoperative interventions according to Clavien-Dindo regardless of presumed causality, acknowledging that definitive attribution is often clinically impossible [35]. The authors proposed organ-specific grading systems to address such liver-specific ambiguities, although this increases complexity and reduces cross-study comparability.

### Clinical Implications

These findings have direct implications for clinical workflow integration and surgical outcomes research. LLM-based systems could automatically propose Clavien-Dindo grades after discharge documentation is completed, functioning as decision-support tools that reduce documentation burden while preserving physician oversight. In the envisioned deployment scenario, the treating clinician would receive an automated grade recommendation directly upon completion of the discharge letter and could either accept or modify the suggested grade before it is permanently recorded in the hospital information system. Thereby preserving clinical authority while reducing manual abstraction effort and contributing to a continuously growing, structured outcomes database. This approach embeds automated grading as a decision-support artifact that augments rather than replaces clinical judgment, consistent with evidence that machine learning–based clinical decision support systems achieve high clinician acceptance when designed to preserve physician oversight [36,37]. To mitigate the risk of high-impact misclassifications in clinical deployment, we propose that document characteristics associated with elevated misclassification risk, such as increased discharge letter length or the presence of ambiguous procedural terminology, should serve as additional flags for prioritized human oversight. For broader adoption in clinical practice, AI-derived classifications will ultimately require structured and interoperable data representations. HL7 FHIR provides a potential framework for integrating such outputs into clinical information systems [23]. As implementation aspects were beyond the scope of this study, further considerations are provided in Figure S3 in Multimedia Appendix 1.

Beyond real-time use, automated grading is particularly well-suited for retrospective research. Large surgical cohorts can be efficiently annotated when complication grading was not performed systematically at the time of care, addressing a long-standing bottleneck in outcomes research. By bridging unstructured clinical narratives and structured metrics, LLMs offer scalable, reproducible quality assessment that addresses interrater variability and practical constraints limiting broader adoption of systematic complication scoring.

### Limitations

Despite overall strong performance, a subset of cases demonstrated significant deviations between LLM predictions and expert assessments, reflecting a fundamental tension in the Clavien-Dindo system: classification depends on the therapy required, yet the clinical significance of laboratory abnormalities without intervention remains subject to interpretation, a judgment often not explicitly documented in discharge letters. More broadly, it should be acknowledged that all classifications, both by human annotators and LLMs, are based exclusively on the information documented in discharge letters, which may not fully reflect the true clinical course. Documentation quality, narrative style, and individual reporting practices may influence the assigned grade, and the system therefore assesses documented rather than verified clinical outcomes. While this does not introduce differential bias in the study design of this study, it represents an inherent limitation of documentation-based outcome classification that should be considered when interpreting results in the context of real-world quality monitoring. Future work should explore richer input representations, including operative notes, progress notes, laboratory values, and imaging reports, to assess the added value of longitudinal documentation for complication grading.

Furthermore, expert annotation was performed by a single senior surgeon, which may introduce individual bias in the assignment of Clavien-Dindo grades. To partially address this limitation, a stratified 10% subset was independently annotated by a second clinician. Complete dual annotation of all 650 cases was not feasible due to the substantial time investment required for expert-level Clavien-Dindo annotation of complex hepatobiliary discharge letters. Disagreements between the 2 annotators were reviewed but did not inform retrospective refinement of the primary annotations, as selectively revising only a subset of the reference standard would have introduced a different form of bias. Formal consensus adjudication was considered but not implemented, as it would have required a third independent expert annotator and represents an avenue for future work. However, interrater agreement was assessed on this subset only and cannot be assumed to be representative. The remaining cases rely solely on single-annotator classification, and observed model-to-expert nonagreement in these cases may therefore partly reflect individual annotator variability rather than true model error. Future studies should incorporate multiple annotators with varying levels of clinical experience to establish a more robust reference standard. In addition, the cases originated from a single care center, reflecting center-specific documentation styles, perioperative pathways, and clinical workflows. Performance may therefore differ in multicenter settings or institutions with alternative reporting standards [38]. At the same time, the 14-year observation period introduces the possibility of temporal changes in documentation practice, which may influence the model performance over time. Multicenter validation studies are essential to assess generalizability and transferability across different clinical contexts.

A further methodological limitation concerns the comparability of open-weight and proprietary model evaluations. Open-weight models were evaluated on the full 650-case dataset, while proprietary models were assessed on a balanced 50-case subset due to cost and access constraints. Although all models were additionally evaluated on the same 50-case subset to enable direct comparison, the difference in evaluation scope limits the interpretability of cross-model conclusions drawn from the full dataset results. All models were evaluated in a zero-shot setting, without task-specific fine-tuning or domain adaptation, thereby limiting performance to intrinsic out-of-the-box reasoning capabilities. Accordingly, the results of this study should be interpreted as a reproducible baseline assessment rather than an optimized deployment scenario. While this choice enables a fair and transparent comparison across models, future work should explore fine-tuning and few-shot prompting, and retrieval-augmented approaches. These methods represent promising strategies for improving performance on challenging grades such as IVa and IVb, where zero-shot performance remained poor across all models. In particular, encoder-only architectures specifically optimized for text classification tasks may represent a more resource-efficient alternative to large generative models and still have high performance. Concurrently, aggregation methods that extend beyond simple majority voting (eg, LLM-as-a-judge frameworks or model councils) have the potential to enhance reliability and clinical alignment by explicitly resolving disagreements, particularly in cases of borderline or rare complication grades. In this context, the robust performance of Ministral-3-8B indicates that further investigation is warranted into SLMs. Encoder-based architectures optimized for text classification may represent a more resource-efficient alternative, with reduced computational cost, inference latency, and ease of integration into clinical IT infrastructures. Nevertheless, the generalizability of these findings is constrained by the size and scope of the dataset. Larger, more diverse, multicenter datasets spanning additional surgical domains are needed to enhance robustness, particularly for rare complication grades. Furthermore, prospective validation in real-world clinical workflows remains a critical next step. Such studies are necessary to evaluate practical utility, assess effects on documentation burden and clinical decision-making, and identify potential biases introduced by automated complication grading systems. Furthermore, although all models were prompted to provide a cited passage as an interpretability basis for their grade assignments, citation accuracy was not formally evaluated and should be considered indicative rather than verified, representing an important direction for future research.

### Conclusion

This study shows that contemporary LLMs can reliably classify postoperative complications according to the Clavien-Dindo system using routine clinical documentation. Open-weight models offer a particularly attractive trade-off by combining competitive accuracy with substantially lower computational demands, while ensemble strategies further enhance robustness. Notably, the performance of the best open-weight models approached the upper bound of human interrater agreement, suggesting that remaining discrepancies between model and expert annotation may partly reflect the inherent variability of clinical grading rather than model limitations alone. Overall, these results highlight the potential of LLMs as a useful tool for supporting surgical complication assessment at scale.

## Data Availability

The dataset used in this study is not publicly available. Individuals or academic organizations interested in utilizing this dataset must submit a detailed request to “Data-Governance@uk-essen.de,” which will be reviewed on a case-by-case basis. We plan to publish the code and make our pipeline available under the repository “UMEssen/CAMEL” on GitHub.

## Appendix

Multimedia Appendix 1 Supplementary material including all additional text boxes, tables, and figures referenced in the manuscript, providing extended methodological details, model-specific performance metrics, prompt templates, and cohort characteristics.

## Abbreviations

CRLM: colorectal liver metastases
FHIR: Fast Healthcare Interoperability Resources
GDPR: General Data Protection Regulation
HCC: hepatocellular carcinoma
ICC: intrahepatic cholangiocarcinoma
ICU: intensive care unit
LLM: large language model
LOS: length of stay
OR: odds ratio
SLM: small language model
