# Supplementary material for: Zero-Shot Classification of Postoperative Complications From Real-World Discharge Letters According to the Clavien-Dindo System Using Large Language Models in Liver Surgery: Comparative Study
Source: J Med Internet Res. 2026 Aug 21;28:e92407. doi: 10.2196/92407 (PMC13545534; doi:10.2196/92407)
Supplement: Supplementary file 1 [file jmir_v28i1e92407_app1.docx]

Kontext:

Du bist ein medizinischer Experte mit Spezialisierung auf postoperative Komplikationen. Du erhältst einen Arztbrief, der Informationen zum postoperativen Verlauf eines Patienten enthalten.

Der Arztbrief enthält Informationen zur durchgeführten Operation und beschreibt den postoperativen Verlauf.

Nur Ereignisse oder Maßnahmen, die im Arztbrief dokumentiert sind und nach Abschluss der Operation aufgetreten sind, dürfen als postoperative Komplikationen bewertet werden.

Der OP-Informationen können dabei helfen, zu erkennen, welche Behandlungen als Standardmaßnahmen zu betrachten sind und welche postoperativen Maßnahmen eine Abweichung bzw. Komplikation darstellen.

Deine Aufgabe ist es, die schwerwiegendste dokumentierte Komplikation nach der Operation zu identifizieren und sie nach der Clavien-Dindo-Klassifikation (Grad 0 bis V) einzuordnen.

Hinweis zur Clavien-Dindo-Klassifikation:

Verwende folgende Definitionen zur Klassifikation:

Grad 0: Keine Komplikation.

Grad I: Abweichung vom normalen postoperativen Verlauf ohne Notwendigkeit pharmakologischer Behandlung oder invasiver Intervention. Zulässig: Antiemetika, Antipyretika, Analgetika, Diuretika, Elektrolyte, Physiotherapie, Wundöffnung am Bett.

Grad II: Komplikation erfordert medikamentöse Behandlung (z. B. Antibiotika, Bluttransfusion, parenterale Ernährung).

Grad IIIa: Intervention ohne Vollnarkose (z. B. Drainage, Endoskopie).

Grad IIIb: Intervention unter Vollnarkose (z. B. Reoperation).

Grad IVa: Lebensbedrohliche Komplikation, intensivmedizinische Überwachung/Behandlung eines Organsystems.

Grad IVb: Lebensbedrohliche Komplikation mit Multiorganversagen.

Grad V: Tod des Patienten aufgrund der Komplikation.

Falls mehrere postoperative Komplikationen auftreten, klassifiziere die schwerwiegendste.

Falls keine Klassifikation möglich ist, gib „Nicht klassifizierbar“ an und erkläre warum.

Standardtherapien (z. B. Schmerztherapie, Thromboseprophylaxe, routinemäßige Antibiose) zählen nicht als Komplikation.

Medikamente oder Therapien, die auf Vorerkrankungen hinweisen und die der Patient möglicherweise bereits vor der Operation erhalten hat und nach der Operation lediglich fortgeführt wurden, stellen keine Komplikation dar.

Bei einem komplikationslosen Verlauf ist Clavien-Dindo Grad 0 zu vergeben.

Bewerte ausschließlich Maßnahmen, die aufgrund eines abnormalen postoperativen Verlaufs erforderlich wurden.

Antwortformat (bitte exakt befolgen):

Clavien-Dindo-Klassifikation: Grad [X]

Begründung: [Kurze medizinisch fundierte Erklärung, warum dieser Grad gewählt wurde. Was ist passiert? Welche Intervention war erforderlich?]

Zitierte Passage: „[Originaltextstelle aus OP-Bericht oder Arztbrief, auf der die Klassifikation basiert]“

Hinweise:

Verwende die Clavien-Dindo-Definition zur Bewertung: Die Klassifikation richtet sich nach der Behandlung, die aufgrund der nach der Operation aufgetretenen Komplikation erforderlich war (z. B. medikamentös, interventionell, chirurgisch, Reanimation, Tod).

Die Sprache der medizinischen Inhalte ist Deutsch. Schreibe die Antwort ebenfalls auf Deutsch.

Antworte nur im vorgegebenen Format. Keine zusätzlichen Erklärungen oder Absätze.

Arztbrief:

Supplement S1: Prompt template used for fine-grained Clavien–Dindo grade prediction in German, as applied to all evaluated models. The prompt instructs the model to classify postoperative complications documented in discharge letters into one of nine Clavien–Dindo grades (0, I, II, IIIa, IIIb, IVa, IVb, V). The prompt was identical across all models and applied in a zero-shot setting without task-specific examples or fine-tuning.

Context:
You are a medical expert specializing in postoperative complications. You are provided with a discharge letter that contains information about a patient’s postoperative course.

The discharge letter includes information about the surgical procedure performed and describes the postoperative course.
Only events or measures that are documented in the discharge letter and occurred after completion of the operation may be evaluated as postoperative complications.
The surgical information can help to identify which treatments should be considered standard measures and which postoperative measures represent a deviation or a complication.

Your task is to identify the most severe documented complication after the operation and to classify it according to the Clavien–Dindo classification (Grade 0 to V).

Note on the Clavien–Dindo classification:
Use the following definitions for classification:

Grade 0: No complication.

Grade I: Any deviation from the normal postoperative course without the need for pharmacological treatment or invasive intervention. Allowed: antiemetics, antipyretics, analgesics, diuretics, electrolytes, physiotherapy, bedside wound opening.

Grade II: Complication requiring pharmacological treatment (e.g., antibiotics, blood transfusion, parenteral nutrition).

Grade IIIa: Intervention without general anesthesia (e.g., drainage, endoscopy).

Grade IIIb: Intervention under general anesthesia (e.g., reoperation).

Grade IVa: Life-threatening complication requiring intensive care monitoring/treatment of a single organ system.

Grade IVb: Life-threatening complication with multiorgan failure.

Grade V: Death of the patient due to the complication.

If multiple postoperative complications occur, classify the most severe one.

If classification is not possible, state “Not classifiable” and explain why.

Standard therapies (e.g., pain management, thrombosis prophylaxis, routine antibiotic therapy) do not count as complications.

Medications or therapies that indicate pre-existing conditions and that the patient may have already received before surgery and that were merely continued after surgery do not constitute a complication.

In the case of an uncomplicated course, Clavien–Dindo Grade 0 should be assigned.

Evaluate only measures that were required due to an abnormal postoperative course.

Response format (please follow exactly):

Clavien–Dindo classification: Grade [X]

Justification: [Brief, medically sound explanation of why this grade was chosen. What happened? What intervention was required?]

Cited passage: “[Original text excerpt from the operative report or discharge letter on which the classification is based]”

Notes:

Use the Clavien–Dindo definition for assessment: The classification is based on the treatment required due to the complication that occurred after the operation (e.g., pharmacological, interventional, surgical, resuscitation, death).

The language of the medical content is German. Write the answer in German as well.

Respond only in the specified format. No additional explanations or paragraphs.

Discharge letter:

Supplement S2: Prompt template used for fine-grained Clavien–Dindo grade prediction; English translation of the original German prompt for reference. The prompt instructs the model to classify postoperative complications documented in discharge letters into one of nine Clavien–Dindo grades (0, I, II, IIIa, IIIb, IVa, IVb, V). The prompt was identical across all models and applied in a zero-shot setting without task-specific examples or fine-tuning.

Kontext:

Du bist ein medizinischer Experte mit Spezialisierung auf postoperative Komplikationen. Du erhältst einen Arztbrief, der Informationen zur Operation und zum postoperativen Verlauf eines Patienten enthält.

Nur Ereignisse oder Maßnahmen, die nach Abschluss der Operation aufgetreten sind und im Arztbrief dokumentiert sind, dürfen als postoperative Komplikationen bewertet werden.

Behandlungen, die als Teil des normalen postoperativen Verlaufs gelten, stellen keine Komplikationen dar.

Deine Aufgabe ist es, die schwerwiegendste dokumentierte postoperative Komplikation zu identifizieren und sie einer der beiden Kategorien A oder B zuzuordnen.

Definition der Kategorien:

Kategorie A – leichte bis moderate postoperative Komplikation

Zu Kategorie A gehören alle Ereignisse oder Maßnahmen, die eine milde bis moderate Abweichung vom normalen postoperativen Verlauf darstellen, darunter:

Keine Komplikation (vollständig komplikationsloser Verlauf)

Maßnahmen ohne spezifische medikamentöse Therapie

(Standardmedikation wie Antiemetika, Analgetika, Antipyretika, Elektrolyte, Diuretika, Physiotherapie oder Wundöffnung am Bett sind erlaubt)

Medikamentöse Therapie aufgrund einer Komplikation, z. B.:

Antibiotikatherapie

Bluttransfusion

Parenterale Ernährung

Interventionen ohne Vollnarkose, z. B.:

Drainageanlage

Punktionz

Endoskopische Intervention ohne Vollnarkose

Kategorie B – schwere postoperative Komplikation

Zu Kategorie B gehören alle Ereignisse, die eine schwere Komplikation darstellen und eine der folgenden Maßnahmen erfordern:

Interventionen unter Vollnarkose, z. B. Reoperation

Lebensbedrohliche Komplikationen mit intensivmedizinischer Überwachung oder Behandlung eines Organsystems

Multiorganversagen

Tod des Patienten infolge der Komplikation

Regeln zur Interpretation:

Falls mehrere Komplikationen auftreten, ordne die schwerwiegendste ein.

Standardtherapien zählen nicht als Komplikation.

Therapien zur Behandlung vorbestehender Erkrankungen, die lediglich fortgeführt werden, zählen nicht als Komplikation.

Wenn keine Bewertung möglich ist, gib „Nicht klassifizierbar“ an und erkläre kurz warum.

Antwortformat (bitte exakt einhalten):

Kategorie: [A oder B]

Begründung:

[Kurze medizinische Erklärung, warum diese Kategorie gewählt wurde. Welche Komplikation trat auf? Welche Behandlung/Intervention war erforderlich?]

Zitierte Passage:

„[Originaltextstelle aus Arztbrief, die die Einordnung begründet]“

Hinweis:

Die Entscheidung richtet sich ausschließlich nach der tatsächlich durchgeführten Behandlung, die aufgrund einer Komplikation erforderlich wurde.

Schreibe die Antwort auf Deutsch.

Keine zusätzlichen Erklärungen außerhalb des Antwortformats.

Arztbrief:

Supplement S3: Prompt template used for binary Clavien–Dindo grade prediction in German, as applied to all evaluated models. The prompt instructs the model to classify postoperative complications documented in discharge letters into two categories: Category A (no or minor complications, Grade 0–IIIa) and Category B (major complications, Grade IIIb–V). The prompt was identical across all models and applied in a zero-shot setting without task-specific examples or fine-tuning.

Context:
You are a medical expert specializing in postoperative complications. You are provided with a discharge letter that contains information about the operation and the patient’s postoperative course.
Only events or measures that occurred after completion of the operation and are documented in the discharge letter may be evaluated as postoperative complications.
Treatments that are considered part of the normal postoperative course do not constitute complications.
Your task is to identify the most severe documented postoperative complication and assign it to one of the two categories A or B.

Definition of the categories:

Category A – mild to moderate postoperative complication
Category A includes all events or measures that represent a mild to moderate deviation from the normal postoperative course, including:

No complication (completely uncomplicated course)

Measures without specific pharmacological therapy
(Standard medication such as antiemetics, analgesics, antipyretics, electrolytes, diuretics, physiotherapy, or bedside wound opening are permitted)

Pharmacological therapy due to a complication, e.g.: Antibiotic therapy, Blood transfusion, Parenteral nutrition

Interventions without general anesthesia, e.g.: Placement of a drain, Puncture, Endoscopic intervention without general anesthesia

Category B – severe postoperative complication
Category B includes all events that represent a severe complication and require one of the following measures:

Interventions under general anesthesia, e.g. reoperation

Life-threatening complications requiring intensive care monitoring or treatment of a single organ system

Multiorgan failure

Death of the patient as a result of the complication

Rules for interpretation:

If multiple complications occur, assign the most severe one.

Standard therapies do not count as complications.

Therapies for the treatment of pre-existing conditions that are merely continued do not count as complications.

If no assessment is possible, state “Not classifiable” and briefly explain why.

Response format (please follow exactly):

Category: [A or B]
Justification:
[Brief medical explanation of why this category was chosen. Which complication occurred? Which treatment/intervention was required?]
Cited passage:
“[Original text excerpt from the discharge letter that justifies the classification]”

Note:
The decision is based exclusively on the treatment that was actually carried out and was required due to a complication.
Write the answer in English.
No additional explanations outside the response format.

Discharge letter:

Supplement S4: Prompt template used for binary Clavien–Dindo grade prediction, as applied to all evaluated models. The prompt instructs the model to classify postoperative complications documented in discharge letters into two categories: Category A (no or minor complications, Grade 0–IIIa) and Category B (major complications, Grade IIIb–V). The original prompt was written in German, reflecting the language of the clinical discharge letters; an English translation is provided for reference. The prompt was identical across all models and applied in a zero-shot setting without task-specific examples or fine-tuning.

**Table S1.** Model specifications for all large language models evaluated in this study, including full model name, number of parameters, context window length, and inference temperature. All models were prompted in a zero-shot setting with temperature set to 0.0 to ensure deterministic and reproducible outputs. Parameter counts and context window lengths for proprietary models (GPT-5.1, Gemini 3 Pro Preview) are not publicly disclosed.

| Model Name | Number of Parameters | Context Window Length | Temperature |
| --- | --- | --- | --- |
| Qwen3-235B-A22B- Instruct-2507-FP8 | 235B | 262 k tokens | 0.0 |
| Llama-3.3-70B- Instruct | 70B | 6k tokens | 0.0 |
| gpt-oss-120b | 120B | 131 k tokens | 0.0 |
| Ministral-3-8B- Instruct-2512 | 8B | 256k tokens | 0.0 |
| gpt-5.1-2025-11-13 | Proprietary | Proprietary | 0.0 |
| gemini-3-pro-preview | Proprietary | Proprietary | 0.0 |

**Table S2.** Cohort characteristics of the balanced, de-identified 50-case subset used for cross-model comparison between open-weight and proprietary models, stratified by Clavien–Dindo grade. For each grade, the number of cases and proportion of female patients, median age with interquartile range (IQR), case distribution across hepatobiliary disease entities, year span of included cases, median discharge letter length in characters and words, and median length of hospital stay are reported. ICC: intrahepatic cholangiocarcinoma; HCC: hepatocellular carcinoma; CRLM: colorectal liver metastases; IQR: interquartile range; LOS: length of stay.

| **Clavien-Dindo Grade** | **0** | **I** | **II** | **IIIa** | **IIIb** | **IVa** | **IVb** | **V** |
| --- | --- | --- | --- | --- | --- | --- | --- | --- |
| **total cases (%)** | 6 (12) | 7 (14) | 5 (10) | 6 (12) | 7 (14) | 6 (12) | 6 (12) | 7 (14) |
| **total female (%)** | 2 (33) | 3 (43) | 1 (20) | 3 (50) | 2 (29) | 2 (33) | 0 (0) | 0 (0) |
| **Median age ([IQR] *in years*)** | 63.5 [56- 73.25] | 67 [61.5-70.5] | 77 [62-81] | 65.5 [61.25- 66.75] | 71 [70.5-73.5] | 65.0 [62-67.25] | 72.0 [67-75.5] | 72.0 [66-75.5] |
| **total cases per entity (%)** | | | | | | | | |
| **ICC**  **HCC**  **klatskin**  **CRLM** | 3 (50)  1 (17)  1 (17)  1 (17) | 3 (43)  1 (14)  2 (29)  1 (14) | 1 (20)  4 (80) | 5 (83)  1 (17) | 1 (14)  2 (29)  4 (57) | 2 (67)  4 (33) | 1 (17)  2 (33)  1 (17)  2 (33) | 1 (14)  3 (43)  3 (43) |
| **year span** | 2012-2023 | 2012-2022 | 2012-2022 | 2012-2022 | 2012-2022 | 2014-2020 | 2017-2023 | 2011-2020 |
| **Median text length ([IQR] *in character)*** | 4557 [3772.5- 5082.75] | 3918 [3471- 5611.5] | 5563 [4605-  7497] | 5473 [4180.5- 6875.75] | 5985 [5651- 7086] | 8419.5 [6806- 9318.25] | 8486 [7162.5- 9709] | 4563 [4001- 6414.5] |
| **Median text length ([IQR] *in words*)** | 549 [439.75- 585.5] | 459 [414- 697] | 665 [517- 930] | 682.5 [501.5- 862.75] | 711 [656.5- 838.5] | 1027 [833- 1078.5] | 1050.5 [847-1215] | 536 [462-740] |
| **Median LOS ([IQR] *in days*)** | 11 [7.25- 14.75] | 11 [9.5- 15.5] | 20 [16-32] | 26.5 [18.75- 33.5] | 21 [17.5- 25.5] | 42.5 [31.5- 64.75] | 94.5 [42.25- 100-25] | 14 [11- 19.5] |

**Supplement S5: Correlation without grade V**

Because Clavien–Dindo Grade V represents postoperative mortality and therefore constitutes a clinical outlier category with fundamentally different trajectories, all correlation analyses were additionally repeated after excluding Grade V cases. Removing this terminal outcome group resulted in stronger associations between complication severity and both length of stay and document length (Spearman’s ρ for document length for characters: 0.41, and for words: ρ=0.40; Spearman’s ρ for LOS: 0.57, for all: p < 0.001 ). These findings suggest that the presence of mortality cases—which often have brief postoperative courses and atypical documentation patterns—attenuates the correlations observed in the full cohort.

**Table S3.** Per-grade precision and recall for fine-grained Clavien–Dindo grade prediction across four open-weight models (Qwen3-235B, Llama-3.3-70B, GPT-OSS-120B, Ministral-3-8B), evaluated on the full 650-case dataset.

| **Grade** |  | **Qwen3-235B** | **Llama 3.3- 70B** | **GPT-OSS 120B** | **Ministral 3- 8B** |
| --- | --- | --- | --- | --- | --- |
| **0** | Precision | **0.938** | 0.944 | 0.919 | 0.891 |
|  | Recall | 0.904 | 0.853 | 0.880 | **0.952** |
| **I** | Precision | 0.634 | 0.549 | 0.633 | **0.763** |
|  | Recall | 0.5 | **0.556** | 0.422 | 0.322 |
| **II** | Precision | 0.483 | 0.412 | **0.529** | 0.476 |
|  | Recall | 0.667 | **0.746** | 0.714 | 0.619 |
| **IIIa** | Precision | 0.563 | **0.771** | 0.607 | 0.627 |
|  | Recall | 0.571 | 0.429 | **0.810** | 0.587 |
| **IIIb** | Precision | 0.727 | 0.707 | **0.795** | 0.764 |
|  | Recall | **0.780** | 0.707 | 0.756 | 0.659 |
| **IVa** | Precision | 0.188 | **0.304** | 0.105 | 0.233 |
|  | Recall | 0.273 | **0.636** | 0.182 | **0.636** |
| **IVb** | Precision | 0.667 | **1.0** | 0.0 | 0.667 |
|  | Recall | **0.333** | 0.167 | 0.0 | **0.333** |
| **V** | Precision | **0.977** | 0.953 | 0.976 | 0.976 |
|  | Recall | **1.0** | 0.976 | 0.976 | 0.952 |

**Table S4.** Omission rate and accuracy for fine-grained Clavien–Dindo grade prediction, reporting the number of “not classifiable” outputs per grade for Ministral-3-8B, overall omission rate, accuracy excluding unclassified cases from the denominator (ACC), and accuracy treating unclassified cases as prediction errors (ACC with all cases).

| **Grade** | **Ministral 3- 8B** |
| --- | --- |
| **0** | 3 |
| **II** | 1 |
| **IIIa** | 1 |
| **Total** | 5 |
| **Omission rate** | 0.8% |
| **ACC** | 0.774 |
| **ACC with all cases** | 0.768 |

**Table S5.** Entity-level classification accuracy for fine-grained Clavien–Dindo grade prediction across four open-weight models (Qwen3-235B, Llama-3.3-70B, GPT-OSS-120B, Ministral-3-8B), evaluated on the full 650-case dataset. Accuracy is reported separately for each hepatobiliary disease entity. HCC: hepatocellular carcinoma; ICC: intrahepatic cholangiocarcinoma; CRLM: colorectal liver metastases; Klatskin: perihilar cholangiocarcinoma.

| Entity | Qwen3 235B | Llama 3.3 70B | GPT-OSS 120B | Ministral 3 8B |
| --- | --- | --- | --- | --- |
| HCC | **0.827** | 0.788 | 0.769 | 0.808 |
| ICC | 0.753 | 0.735 | 0.753 | **0.765** |
| CRLM | **0.832** | **0.832** | 0.823 | 0.823 |
| Klatskin | 0.735 | 0.687 | **0.768** | 0.725 |
| Other | 0.5 | 0.75 | 0.625 | **0.857** |

**Table S6.** Model performance stratified by year of discharge letter (2010–2024) in predicting the finegrading Clavien–Dindo grade. For each calendar year, overall accuracy (ACC) and weighted F1-score (F1) are reported for all four open-weight models evaluated on the full dataset. Years with low case counts should be interpreted with caution. ACC: overall accuracy, F1: weighted F1-score, calculated as the weighted average of per-class F1-scores proportional to class frequency, accounting for the class imbalance inherent in the annual case distributions.

| Year | N | Qwen3 235B | | Llama 3.3 70B | | GPT-OSS 120B | | Ministral 3 8B | |
| --- | --- | --- | --- | --- | --- | --- | --- | --- | --- |
|  |  | **ACC** | **F1** | **ACC** | **F1** | **ACC** | **F1** | **ACC** | **F1** |
| 2010 | 4 | **0.5** | **0.5** | **0.5** | **0.5** | **0.5** | **0.5** | **0.5** | **0.5** |
| 2011 | 10 | **0.9** | **0.9** | 0.8 | 0.754 | 0.8 | 0.787 | 0.8 | 0.846 |
| 2012 | 47 | **0.851** | **0.844** | 0.766 | 0.744 | 0.787 | 0.779 | 0.787 | 0.802 |
| 2013 | 51 | 0.824 | 0.827 | 0.745 | 0.750 | **0.843** | **0.851** | 0.78 | 0.775 |
| 2014 | 62 | 0.758 | 0.751 | 0.710 | 0.681 | **0.823** | **0.813** | 0.774 | 0.779 |
| 2015 | 50 | 0.72 | 0.708 | **0.74** | 0.735 | 0.7 | 0.676 | **0.74** | **0.756** |
| 2016 | 45 | 0.8 | 0.786 | 0.778 | 0.783 | **0.822** | **0.816** | 0.778 | 0.787 |
| 2017 | 53 | **0.811** | **0.834** | 0.774 | 0.803 | 0.660 | 0.678 | 0.755 | 0.793 |
| 2018 | 25 | 0.64 | 0.608 | **0.68** | 0.651 | 0.64 | 0.644 | **0.68** | **0.676** |
| 2019 | 45 | **0.822** | 0.806 | 0.711 | 0.691 | **0.822** | **0.836** | 0.778 | 0.767 |
| 2020 | 49 | **0.776** | **0.776** | 0.735 | 0.734 | 0.755 | 0.762 | 0.771 | 0.766 |
| 2021 | 40 | **0.8** | **0.808** | 0.675 | 0.646 | 0.75 | 0.740 | 0.75 | 0.748 |
| 2022 | 48 | 0.708 | 0.734 | **0.771** | 0.769 | **0.771** | **0.795** | 0.75 | 0.750 |
| 2023 | 78 | 0.795 | 0.780 | **0.821** | **0.809** | 0.782 | 0.778 | 0.805 | 0.799 |
| 2024 | 43 | 0.721 | 0.733 | 0.767 | 0.751 | **0.837** | 0.844 | **0.837** | **0.866** |

**Figure S1.** Confusion matrices for fine-grained Clavien–Dindo grade prediction stratified by complication severity group across four open-weight models (Qwen3-235B, Llama-3.3-70B, GPT-OSS-120B, Ministral-3-8B), evaluated on the full 650-case dataset. Cases are grouped into minor complications (Grade ≤IIIa) and major complications (Grade ≥IIIb). Rows represent the expert-annotated reference labels; columns represent model predictions.

**Figure S2.** Confusion matrices for binary Clavien–Dindo grade prediction across four open-weight models (Qwen3-235B, Llama-3.3-70B, GPT-OSS-120B, Ministral-3-8B), evaluated on the full 650-case dataset. Rows represent the expert-annotated reference labels; columns represent model predictions. Category A: Grade 0–IIIa; Category B: Grade IIIb–V.

**Table S7.** Omission rate and adjusted accuracy for binary Clavien–Dindo grade prediction across four open-weight models, evaluated on the full 650-case dataset. For each model, the number of 'not classifiable' outputs per binary category (A: Grade 0–IIIa; B: Grade IIIb–V) and the overall omission rate are reported. Accuracy is provided under two calculation methods: excluding unclassified cases from the denominator (ACC) and treating unclassified cases as prediction errors (ACC with all cases). ACC: accuracy.

|  | **Qwen3 235B** | **Llama 3.3 70B** | **gpt-oss 120B** | **Ministral 3 8B** |
| --- | --- | --- | --- | --- |
| **Category A** | 3 | 0 | 1 | 36 |
| **Category B** | 0 | 0 | 1 | 0 |
| **Total** | 3 | 0 | 2 | 36 |
|  |  |  |  |  |
| **Omission rate** | 0.5% | 0.0% | 0.3% | 5.5% |
| **ACC** | 0.943 | 0.942 | 0.943 | 0.928 |
| **ACC with all cases** | 0.938 | 0.942 | 0.94 | 0.877 |

**Table S8.** Entity-level classification accuracy for binary Clavien–Dindo grade prediction (Category A: Grade 0–IIIa; Category B: Grade IIIb–V) across four open-weight models, evaluated on the full 650-case dataset. Accuracy is reported separately for each hepatobiliary disease entity. HCC: hepatocellular carcinoma; ICC: intrahepatic cholangiocarcinoma; CRLM: colorectal liver metastases; Klatskin: perihilar cholangiocarcinoma.

| Entity | Qwen3 235B | Llama 3.3 70B | gpt-oss 120B | Ministral 3 8B |
| --- | --- | --- | --- | --- |
| HCC | 0.955 | **0.962** | 0.949 | 0.951 |
| ICC | 0.938 | **0.951** | 0.944 | 0.928 |
| CRLM | 0.965 | 0.982 | **0.991** | 0.981 |
| klatskin | **0.905** | 0.896 | 0.900 | 0.883 |
| Other | **1.0** | **1.0** | 0.875 | **1.0** |

**Table S9.** Model performance stratified by year of discharge letter (2010–2024) in predicting the binary Clavien–Dindo grade. For each calendar year, overall accuracy (ACC) and weighted F1-score (F1) are reported for all four open-weight models evaluated on the full dataset. Years with low case counts should be interpreted with caution. ACC: overall accuracy, F1: weighted F1-score, calculated as the weighted average of per-class F1-scores proportional to class frequency, accounting for the class imbalance inherent in the annual case distributions.

| Year | N | Qwen3 235B | | Llama 3.3 70B | | GPT-OSS 120B | | Ministral 3 8B | |
| --- | --- | --- | --- | --- | --- | --- | --- | --- | --- |
|  |  | **ACC** | **F1** | **ACC** | **F1** | **ACC** | **F1** | **ACC** | **F1** |
| 2010 | 4 | **0.75** | **0.643** | **0.75** | **0.643** | **0.75** | **0.643** | 0.5 | 0.5 |
| 2011 | 10 | 0.8 | 0.874 | **0.9** | **0.886** | **0.9** | **0.886** | 0.8 | 0.874 |
| 2012 | 47 | 0.936 | 0.929 | **0.957** | **0.954** | 0.936 | 0.929 | 0.932 | 0.924 |
| 2013 | 51 | **0.94** | **0.941** | 0.902 | 0.904 | 0.922 | 0.925 | 0.870 | 0.870 |
| 2014 | 62 | 0.951 | 0.948 | 0.952 | 0.948 | **0.968** | **0.966** | 0.883 | 0.870 |
| 2015 | 50 | 0.96 | 0.958 | **0.98** | **0.979** | 0.92 | 0.912 | 0.938 | 0.932 |
| 2016 | 45 | **0.933** | **0.929** | **0.933** | **0.929** | **0.933** | **0.929** | 0.930 | 0.926 |
| 2017 | 53 | 0.943 | 0.944 | 0.925 | 0.926 | 0.943 | 0.945 | **0.962** | **0.962** |
| 2018 | 25 | 0.84 | 0.84 | 0.88 | 0.885 | **0.92** | **0.928** | 0.84 | 0.84 |
| 2019 | 45 | **0.978** | **0.977** | 0.956 | 0.953 | **0.978** | **0.977** | 0.976 | 0.975 |
| 2020 | 49 | 0.939 | 0.938 | **0.959** | **0.958** | 0.875 | 0.870 | 0.930 | 0.929 |
| 2021 | 40 | **0.975** | **0.974** | 0.925 | 0.916 | 0.95 | 0.946 | 0.95 | 0.946 |
| 2022 | 48 | 0.896 | 0.887 | 0.958 | 0.957 | 0.958 | 0.958 | **0.978** | **0.978** |
| 2023 | 78 | 0.962 | 0.961 | **0.974** | **0.974** | **0.974** | **0.974** | 0.959 | 0.957 |
| 2024 | 43 | 0.953 | **0.950** | 0.907 | 0.900 | **0.976** | 0.75 | 0.9 | 0.893 |

**Table S10.** Classification performance for binary Clavien–Dindo grading across four open-weight large language models, evaluated on the full 650-case dataset. Binary categories are defined as Category A (no or minor complications, Clavien–Dindo Grade 0–IIIa) and Category B (major complications, Clavien–Dindo Grade IIIb–V). Per-category metrics include precision, recall, and F1-score. Overall performance is summarized as macro-averaged F1-score, weighted F1-score, and overall accuracy. Model-to-expert agreement is quantified as Cohen's kappa (κ), calculated on the stratified 65-case subset (10% of the full dataset) used for inter-rater reliability assessment, allowing direct comparison with the human inter-rater benchmark (κ = 0.843). IRR: Inter-Rater Reliability.

|  |  | **Qwen3-235B** | **Llama 3.3-70B** | **GPT-OSS 120B** | **Ministral 3-8B** |
| --- | --- | --- | --- | --- | --- |
| **Category A** | **Precision** | **0.985** | 0.983 | 0.979 | 0.984 |
|  | **Recall** | 0.945 | 0.946 | **0.953** | 0.930 |
|  | **F1-Score** | 0.965 | 0.965 | **0.966** | 0.956 |
| **Category B** | **Precision** | 0.754 | 0.758 | **0.772** | 0.719 |
|  | **Recall** | **0.92** | 0.91 | 0.889 | **0.92** |
|  | **F1-Score** | **0.829** | 0.827 | 0.826 | 0.807 |
| **Overall** | **macro F1** | **0.897** | 0.896 | 0.896 | 0.882 |
|  | **weighted F1** | 0.944 | 0.944 | **0.945** | 0.932 |
|  | **Accuracy** | 0.941 | 0.942 | **0.943** | 0.928 |
| **10% IRR-Subset** | **Cohen’s Kappa** | **0.877** | 0.846 | 0.845 | 0.815 |

**Table S11.** Grade-level and overall classification performance for fine-grained Clavien–Dindo grade prediction across four open-weight models (Qwen3-235B, Llama-3.3-70B, GPT-OSS-120B, Ministral-3-8B) and two proprietary models (GPT-5.1, Gemini 3 Pro Preview), all evaluated on the same balanced, de-identified 50-case subset in a zero-shot setting. For each Clavien–Dindo grade, accuracy and F1-score. Overall performance is summarized as overall accuracy, macro-averaged F1-score, and weighted F1-score. ACC: accuracy.

| Grade | Qwen3 235B | | Llama 3.3 70B | | gpt-oss 120B | | Ministral3 8b | | GPT 5.1 | | Gemini 3 Pro preview | |
| --- | --- | --- | --- | --- | --- | --- | --- | --- | --- | --- | --- | --- |
|  | ACC | F1 | ACC | F1 | ACC | F1 | ACC | F1 | ACC | F1 | ACC | F1 |
| 0 | 0.833 | 0.833 | 0.833 | 0.909 | 0.667 | 0.727 | **1.0** | **0.923** | 0.833 | 0.833 | **1.0** | **0.923** |
| I | **1.0** | **1.0** | 0.857 | 0.857 | 0.857 | 0.857 | 0.714 | 0.833 | **1.0** | 0.933 | **1.0** | 0.875 |
| II | 0.6 | 0.6 | **0.8** | 0.615 | 0.6 | 0.545 | 0.6 | 0.6 | 0.6 | **0.667** | 0.6 | 0.6 |
| IIIa | 0.5 | 0.462 | 0.333 | 0.364 | **0.833** | 0.714 | 0.5 | 0.429 | **0.833** | 0.714 | **0.833** | **0.833** |
| IIIb | **0.857** | 0.75 | 0.714 | 0.667 | 0.714 | 0.588 | 0.571 | 0.571 | 0.714 | 0.714 | **0.857** | **0.800** |
| IVa | 0.333 | 0.364 | **0.667** | 0.533 | 0.167 | 0.167 | **0.667** | 0.571 | **0.667** | **0.667** | 0.333 | 0.444 |
| IVb | 0.333 | 0.444 | 0.167 | 0.286 | 0.0 | 0.0 | 0.333 | 0.5 | **0.5** | **0.667** | **0.5** | 0.6 |
| V | **1.0** | **0.933** | 0.857 | 0.857 | 0.857 | 0.857 | **1.0** | **0.933** | **1.0** | **0.933** | **1.0** | **0.933** |
| Overall Accuracy: | | | | | | | | | | | | |
|  | 0.7 | | 0.66 | | 0.6 | | 0.68 | | **0.78** | | **0.78** | |
| macro averaged F1-Score: | | | | | | | | | | | | |
|  | 0.673 | | 0.636 | | 0.557 | | 0.670 | | **0.766** | | 0.751 | |
| weighted averaged F1-Score: | | | | | | | | | | | | |
|  | 0.688 | | 0.646 | | 0.570 | | 0.678 | | **0.774** | | 0.761 | |

**Table S12.** Entity-level classification accuracy for fine-grained Clavien–Dindo grade prediction across four open-weight models (Qwen3-235B, Llama-3.3-70B, GPT-OSS-120B, Ministral-3-8B) and two proprietary models (GPT-5.1, Gemini 3 Pro Preview), all evaluated on the balanced 50-case subset. Accuracy is reported separately for each hepatobiliary disease entity. HCC: hepatocellular carcinoma; ICC: intrahepatic cholangiocarcinoma; CRLM: colorectal liver metastases; Klatskin: perihilar cholangiocarcinoma.

| Entity | Qwen3 235B | Llama 3.3 70B | GPT-OSS120B | Ministral 3 8B | GPT 5.1 | Gemini 3 Pro preview |
| --- | --- | --- | --- | --- | --- | --- |
| HCC | **0.8** | 0.6 | 0.4 | 0.6 | **0.8** | **0.8** |
| ICC | 0.636 | 0.727 | 0.727 | 0.727 | **0.818** | 0.727 |
| CRLM | **1.0** | 0.8 | 0.6 | 0.8 | **1.0** | 0.8 |
| klatskin | 0.625 | 0.625 | 0.625 | 0.667 | 0.708 | **0.792** |

**Table S13.** Classification accuracy for fine-grained Clavien–Dindo grade prediction stratified by complication severity group across four open-weight models (Qwen3-235B, Llama-3.3-70B, GPT-OSS-120B, Ministral-3-8B) and two proprietary models (GPT-5.1, Gemini 3 Pro Preview), all evaluated on the balanced 50-case subset. Results are reported separately for minor complications (Grade ≤IIIa) and major complications (Grade ≥IIIb).

| Grade | Qwen3 235B | Llama 3.3 70B | GPT-OSS120B | Ministral3 8B | GPT 5.1 | Gemini 3 Pro Preview |
| --- | --- | --- | --- | --- | --- | --- |
| <= IIIa | 0.75 | 0.708 | 0.75 | 0.708 | 0.833 | **0.875** |
| >=IIIb | 0.654 | 0.615 | 0.462 | 0.654 | **0.731** | 0.692 |

**Table S14.** Classification performance for binary Clavien–Dindo grade prediction across four open-weight models (Qwen3-235B, Llama-3.3-70B, GPT-OSS-120B, Ministral-3-8B) and two proprietary models (GPT-5.1, Gemini 3 Pro Preview), all evaluated on the balanced 50-case subset. Per-category metrics include precision, recall, and F1-score. Overall performance is summarized as macro-averaged F1-score, weighted F1-score, and overall accuracy. Category A: Grade 0–IIIa; Category B: Grade IIIb–V.

|  |  | Qwen3 235B | Llama 3.3 70B | GPT-OSS120B | Ministral 3 8B | GPT 5.1 | Gemini 3 Pro Preview |
| --- | --- | --- | --- | --- | --- | --- | --- |
| **Category A** | **Precision** | **0.957** | 1.0 | 0.92 | 0.952 | 0.96 | 0.92 |
|  | **Recall** | 0.917 | 0.875 | **0.958** | 0.833 | 1.0 | 0.958 |
|  | **F1-Score** | 0.936 | 0.933 | **0.939** | 0.889 | 0.980 | 0.939 |
| **Category B** | **Precision** | 0.926 | 0.897 | **0.96** | 0.862 | 1.0 | 0.96 |
|  | **Recall** | **0.962** | 1.0 | 0.923 | **0.962** | **0.962** | **0.923** |
|  | **F1-Score** | **0.943** | 0.945 | 0.941 | 0.909 | 0.980 | 0.941 |
| **Overall** | **macro F1** | **0.940** | 0.940 | 0.940 | 0.900 | 0.980 | 0.940 |
|  | **weighted F1** | 0.940 | 0.940 | **0.940** | 0.899 | 0.980 | 0.940 |
|  | **Accuracy** | 0.94 | 0.94 | 0.94 | 0.90 | **0.98** | 0.94 |

**Table S15.** Entity-level classification accuracy for binary Clavien–Dindo grade prediction (Category A: Grade 0–IIIa; Category B: Grade IIIb–V) across four open-weight models (Qwen3-235B, Llama-3.3-70B, GPT-OSS-120B, Ministral-3-8B) and two proprietary models (GPT-5.1, Gemini 3 Pro Preview), all evaluated on the balanced 50-case subset. Accuracy is reported separately for each hepatobiliary disease entity. HCC: hepatocellular carcinoma; ICC: intrahepatic cholangiocarcinoma; CRLM: colorectal liver metastases; Klatskin: perihilar cholangiocarcinoma.

| Entity | Qwen3 235B | Llama 3.3 70B | GPT-OSS 120B | Ministral 3 8B | GPT 5.1 | Gemini 3 Pro Preview |
| --- | --- | --- | --- | --- | --- | --- |
| HCC | **1.0** | 0.9 | 0.9 | 0.9 | **1.0** | 0.9 |
| ICC | **1.0** | **1.0** | **1.0** | **1.0** | **1.0** | **1.0** |
| CRLM | 0.8 | 0.8 | **1.0** | 0.8 | **1.0** | **1.0** |
| klatskin | 0.917 | **0.958** | 0.917 | 0.875 | **0.958** | 0.917 |

**Table S16.** Relative proportion of absolute deviation from Clavien–Dindo grades shown per grade between model predictions and expert annotations for four open-weight models.

| **Grade** | **Absolute deviation** | **Qwen3 235B** | **Llama 3.3 70B** | **GPT-OSS 120B** | **Ministral 3 8B** |
| --- | --- | --- | --- | --- | --- |
| **0** | 1 | 68.75% | 63.27% | 52.5% | 46.15% |
| **0** | 2 | 28.12% | 24.49% | 30% | 30.77% |
| **0** | 3 |  |  | 7.5% |  |
| **0** | 4 | 3.12% | 10.2% | 2.5% | 7.69% |
| **0** | 5 |  | 2.04% | 7.5% | 15.38% |
| **I** | 1 | 71.11% | 90% | 71.15% | 83.61% |
| **I** | 2 | 20% | 2.5% | 23.08% | 4.92% |
| **I** | 3 |  |  | 1.92% |  |
| **I** | 4 | 8.89% | 7.5% | 3.85% | 11.48% |
| **II** | 1 | 61.9% | 62.5% | 50% | 39.13% |
| **II** | 2 | 28.57% | 31.25% | 27.78% | 34.78% |
| **II** | 3 | 9.52% | 6.25% | 16.67% | 26.09% |
| **II** | 4 |  |  | 5.56% |  |
| **IIIa** | 1 | 61.54% | 75% | 25% | 56% |
| **IIIa** | 2 | 19.23% | 19.44% | 25% | 24% |
| **IIIa** | 3 | 19.23% | 5.56% | 50% | 20% |
| **IIIb** | 1 | 66.67% | 58.33% | 70% | 78.57% |
| **IIIb** | 2 | 33.33% | 41.67% | 20% | 21.43% |
| **IIIb** | 4 |  |  | 10% |  |
| **IVa** | 1 | 50% | 50% | 22.22% | 25% |
| **IVa** | 2 | 50% | 25% | 55.56% | 50% |
| **IVa** | 3 |  | 25% | 22.22% | 25% |
| **IVb** | 1 | 75% | 80% | 66.67% | 75% |
| **IVb** | 2 | 25% | 20% | 33.33% | 25% |
| **V** | 1 |  |  |  | 50% |
| **V** | 2 |  | 100% | 100% |  |
| **V** | 8 |  |  |  | 50% |

**Table S17.** Relative proportion of absolute deviation of Clavien–Dindo grades between model predictions and expert annotations for four open-weight models and two proprietary models on the deidentified subset of 50 cases.

| Deviation absolute | Qwen3 235B | Llama 3.3 70B | GPT-OSS 120B | Ministral 3 8B | GPT 5.1 | Gemini 3 Pro Preview |
| --- | --- | --- | --- | --- | --- | --- |
| 1 | 66.67% | 70.59% | 45% | 68.75% | 54.55% | 54.55% |
| 2 | 26.67% | 29.41% | 35% | 18.75% | 27.27% | 27.27% |
| 3 | 6.67% |  | 15% | 12.5% | 18.18% | 18.18% |
| 4 |  |  | 5% |  |  |  |

**Table S18.** Relative proportion of absolute deviation from Clavien–Dindo grades shown per grade between model predictions and expert annotations for four open-weight models and two proprietary models on the deidentified subset of 50 cases.

| **Grade** | **Absolute deviation** | **Qwen3 235B** | **Llama 3.3 70B** | **GPT-OSS 120B** | **Ministral 3 8B** | **GPT 5.1** | **Gemini 3 Pro Preview** |
| --- | --- | --- | --- | --- | --- | --- | --- |
| **0** | 1 |  | 100% | 50% |  | 100% |  |
| **0** | 2 | 100% |  | 50% |  |  |  |
| **I** | 1 |  |  |  | 50% |  |  |
| **I** | 2 |  | 100% |  | 50% |  |  |
| **I** | 3 |  |  | 100% |  |  |  |
| **II** | 1 | 100% | 100% | 50% | 50% | 50% | 100% |
| **II** | 2 |  |  |  |  |  |  |
| **II** | 3 |  |  |  | 50% | 50% |  |
| **II** | 4 |  |  | 50% |  |  |  |
| **IIIa** | 1 | 33.33% | 75% |  | 66.67% |  |  |
| **IIIa** | 2 | 33.33% | 25% |  |  |  |  |
| **IIIa** | 3 | 33.33% |  | 100% | 33.33% | 100% | 100% |
| **IIIb** | 1 | 100% | 50% | 50% | 100% | 50% |  |
| **IIIb** | 2 |  | 50% | 50% |  | 50% | 100% |
| **IVa** | 1 | 75% | 100% | 40% | 50% | 50% | 50% |
| **IVa** | 2 | 25% |  | 40% | 50% | 50% | 25% |
| **IVa** | 3 |  |  | 20% |  |  | 25% |
| **IVb** | 1 | 75% | 80% | 66.67% | 75% | 66.67% | 66.67% |
| **IVb** | 2 | 25% | 20% | 33.33% | 25% | 33.33% | 33.33% |
| **V** | 1 |  |  |  |  |  |  |
| **V** | 2 |  | 100% | 100% |  |  |  |

 **Figure S3.** FHIR-based workflow for integrating LLM-derived Clavien–Dindo classifications into clinical systems. Schematic illustration of a standards-based workflow for integrating model-generated Clavien–Dindo classifications into clinical information systems. Model inference metadata and contextual information are captured using the FHIR Provenance resource, which references the generated Observation representing the Clavien–Dindo classification. The Observation references the underlying clinical document source via DocumentReference. The interoperable HL7 FHIR (R4) standard enables seamless integration into existing electronic health record systems, where classifications can be used as clinical decision-support artifacts. Created with BioRender.
